# Human *C*. *difficile* toxin–specific memory B cell repertoires encode poorly neutralizing antibodies

**DOI:** 10.1172/jci.insight.138137

**Published:** 2020-08-20

**Authors:** Hemangi B. Shah, Kenneth Smith, Edgar J. Scott, Jason L. Larabee, Judith A. James, Jimmy D. Ballard, Mark L. Lang

**Affiliations:** 1 Department of Microbiology and Immunology, University of Oklahoma Health Sciences Center (OUHSC),; 2 Arthritis and Clinical Immunology, Oklahoma Medical Research Foundation, and; 3 Departments of Medicine and Pathology, OUHSC, University of Oklahoma, Oklahoma City, Oklahoma, USA.

**Keywords:** Immunology, Infectious disease, B cells, Bacterial infections, Memory

## Abstract

*Clostridioides difficile* is a leading cause of nosocomial infection responsible for significant morbidity and mortality with limited options for therapy. Secreted *C*. *difficile* toxin B (TcdB) is a major contributor to disease pathology, and select TcdB-specific Abs may protect against disease recurrence. However, the high frequency of recurrence suggests that the memory B cell response, essential for new Ab production following *C*. *difficile* reexposure, is insufficient. We therefore isolated TcdB-specific memory B cells from individuals with a history of *C*. *difficile* infection and performed single-cell deep sequencing of their Ab genes. Herein, we report that TcdB-specific memory B cell–encoded antibodies showed somatic hypermutation but displayed limited isotype class switch. Memory B cell–encoded mAb generated from the gene sequences revealed low to moderate affinity for TcdB and a limited ability to neutralize TcdB. These findings indicate that memory B cells are an important factor in *C*. *difficile* disease recurrence.

## Abstract 2

The *C. difficile* toxin-specific human memory B cell repertoire encodes low-affinity, non-neutralizing antibodies.

## Graphical abstract

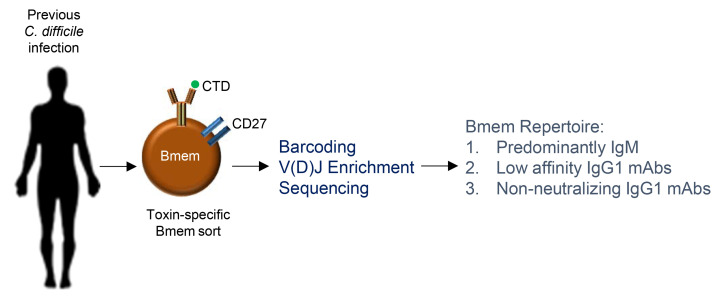

## Introduction

*Clostridioides difficile* is responsible for almost half a million infections and 30,000 deaths in the US annually (1), and it is a significant global health issue (2–5). *C*. *difficile* colonization of the large intestine results in symptoms ranging from diarrhea to life-threatening pseudomembranous colitis, sepsis, and even death (6–11). The causes of *C*. *difficile*–associated mortality are not entirely clear, but case reports suggest that systemic sequelae of the disease are contributory (12). Systemic complications of *C*. *difficile* reported to date include hepatic abscesses (13), ascites (14), pleural effusion and acute respiratory distress (15, 16), and sepsis and multiorgan failure (10).

The enteric and systemic pathology associated with *C*. *difficile* infection (CDI) is attributable to secreted toxins known as *C*. *difficile* toxin A (TcdA) and *C*. *difficile* toxin B (TcdB) (17–19). These toxins enter target cells and glucosylate Rho GTPases to facilitate broad cellular damage (20, 21). Blood-borne TcdA and TcdB can be detected in some patients and are toxic to target cells in vitro (22). However, TcdA-negative strains can also be highly virulent (19, 23), and although there is a recent report of disease associated with a TcdB-negative strain (24), it is clear that TcdB is a major driver of disease. TcdB has systemic toxicity in several animal species (25–28), supporting the observations of systemic pathology in patients.

There are several distinct ribotypes and strains of pathogenic *C*. *difficile* that cause disease of varying severity (29). Infection with a hypervirulent *C*. *difficile* strain such as the NAP1/BI/027 (ribotype 027) is associated with more severe disease than a historical strain such as VPI-10463 (ribotype 003) (30–32). Mutation of TcdB is likely to contribute to differences in disease severity. Although NAP1/BI/027 toxin B (TcdB2) and VPI-10463 toxin B (TcdB1) share 92% sequence identity and are similarly immunogenic (33), TcdB2 is more cytotoxic than TcdB1 (28).

As many as 30% of individuals with an initial CDI will suffer from disease recurrence (34). There are several risk factors for recurrence, including antibiotic use, advanced age, immune response, and the *C*. *difficile* strains to which patients are exposed (35–39). Recurrent CDI is characterized by regrowth of bacteria that have survived antibiotic therapy or by reinfection with *C*. *difficile*, and each recurrence increases the probability of further episodes (40). Recurrence is associated with progressively worsening pathology and increasing mortality (41).

*C. difficile* recurrence indicates that an initial infection failed to adequately immunize the individual and confer protection against subsequent infection. Indeed, patients with higher anti–TcdA and –TcdB serum IgG titers have lower rates of recurrence, and TcdB-specific IgG is the best known correlate of protection against *C*. *difficile* (37, 42–45). For example, in 2 independent studies of patients with CDI, recruiting 99 and 61 patients, respectively, high serum titers of TcdB-binding and/or -neutralizing IgG were associated with a lower rate of disease recurrence (43, 45). Bacterial load during infection correlates directly with age and inversely with TcdB-neutralizing IgG titers (46). There is also indirect evidence for protective humoral immunity. CDI risk is increased in HIV-infected individuals with declining CD4^+^ Th cell counts (47) and in immunosuppressed organ transplant recipients (48). The quality of the IgG response is also important for protection — for example, the TcdB-neutralizing FDA-approved IgG mAb bezlotoxumab binds TcdB with high affinity (49). In a clinical trial, of 200 patients (101 on mAb therapy and 99 on placebo), recurrence was cut by approximately 80% (50). In 2 subsequent double-blind phase III trials of 2655 patients, recurrence was cut by approximately 60% (51). The binding affinity of mAbs to TcdB has only been examined in the context of therapeutic mAbs thus far and needs to be evaluated for Abs from past CDI patients.

Despite the clear association between TcdB-neutralizing IgG and disease protection, B cell memory following CDI is not well characterized, and its consequences for recurrent infection are poorly defined. Antigen-activated B cells can differentiate into short- or long-lived Ab–secreting plasma cells or into memory B (Bmem) cells (reviewed in refs. 52, 53). Restimulation of Bmem cells with booster vaccines or repeat infections can drive their differentiation into new Ab-secreting plasma cells with the added benefit of speed, increased magnitude, prior isotype switch, and somatic hypermutation (SHM) to generate high-affinity Ab.

Weak toxin-specific Bmem cell responses in individuals with CDI were demonstrated previously by our lab and others (33, 54). This warrants a detailed analysis of the Bmem cell–encoded Ab to identify the underlying defects that may prevent an adequate response. We therefore profiled the TcdB-specific Bmem cell repertoire in individuals who had a prior CDI. These individuals self-reported having been diagnosed with CDI, with 1 requiring hospitalization due to CDI. The carboxy-terminal domain (CTD) of TcdB consists of combined repetitive oligopeptides (CROPs) that are known to contain neutralizing epitopes (55, 56). Although infection with strain VPI-10463 is unlikely, the Abs to TcdB1 and TcdB2 are highly cross-reactive (57). We therefore used fluorophore-conjugated CTD (amino acids 1651–2366) of TcdB1 to identify specific Bmem cells in the PBMC of individuals with a history of CDI. Using single-cell barcoding and deep sequencing of bulk-sorted CTD-specific Bmem cells, we generated several hundred complete Ab gene sequences for 3 individuals. Analysis of the Ab features revealed a low degree of isotype switch, with IgM dominating the repertoires. The IgG-encoding Bmem cells demonstrated a high degree of SHM. The IgM, IgA, and IgG repertoires were also dominated by unique clones, indicating very limited clonal expansion. The IgG1 gene sequences were expressed in vitro, resulting in production of 49 full-length, intact IgG1 mAbs. While 50% of the mAbs showed demonstrable binding to TcdB1 and TcdB2, only 1 mAb neutralized TcdB2 in vitro. These data show that the Bmem cell repertoire following CDI encodes affinity-matured Abs that are likely to provide limited protection against TcdB-driven pathogenesis. These results may contribute to an explanation for recurrent disease following CDI.

## Results

### Detection of CTD-specific Bmem cells in individuals with a history of CDI and preparation for repertoire analysis.

**Figure 1:**
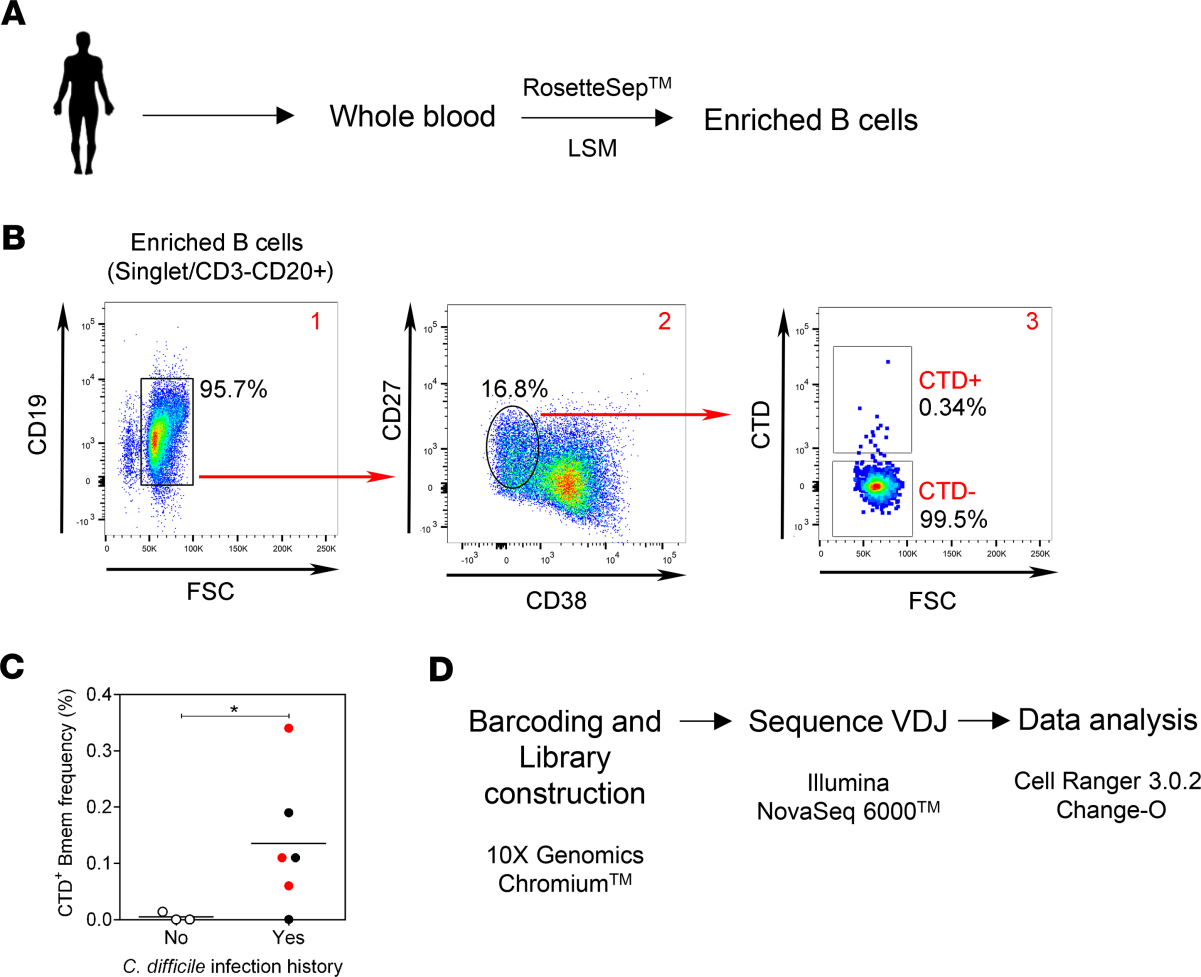
Isolation, sequencing, and repertoire analysis of CTD-specific Bmem cells. (**A**) Blood samples were obtained from subjects with a history of CDI and used as a source of enriched B cells. (**B**) Enriched B cells were labeled with a cocktail of fluorochrome-conjugated mAbs and Alexa 488–conjugated CTD as described in Methods. Pseudocolor plots 1 through 3 depict the gating strategy, allowing identification and sorting of CD19^+^CD20^+^CD27^+^CD38^–^CTD^+^ cells and CD19^+^CD20^+^CD27^+^CD38^–^CTD^–^ cells. Data shown are from subject 1008. (**C**) Shows frequency of CTD^+^ Bmem in 3 healthy controls versus 6 previously infected subjects. The subjects included in the single-cell analysis are denoted by red symbols: subjects 1008 (0.34%), 1009 (0.06%), and 1013 (0.11%). Black dots denote other subjects recruited: subjects 1012 (0%), 1015 (0.11%), and 1018 (0.19%). The line indicates the mean. A 2-tailed unpaired *t* test with Welch’s correction was applied to determine statistical significance in the differences observed (*P < 0.05). (**D**) Sorted CTD^+^ and CTD^–^ Bmem cells were processed as depicted and described in Methods, resulting in the generation of individually barcoded and fully sequenced V(D)J regions for each Bmem cell. FASTA files were generated using the Cell Ranger 3.0.2 pipeline, and the Change-O toolkit was used to analyze the data.

Blood samples were collected from study participants described in Supplemental Table 1 (supplemental material available online with this article; https://doi.org/10.1172/jci.insight.138137DS1) before enrichment of total B cells (Figure 1A). Flow cytometry was performed to identify singlet CD3^–^CD19^+^CD20^+^CD27^+^CD38^–^CTD^+^ Bmem cells and CD3^–^CD19^+^CD20^+^CD27^+^CD38^–^CTD^–^ Bmem cells (Figure 1B). CTD-binding Bmem cells were typically undetectable or present at very low frequencies in individuals who had no known history of CDI (Figure 1C). Therefore, populations of CTD-specific Bmem cells could readily be detected and distinguished from Bmem cells of other specificities. CTD^+^ and CTD^–^ Bmem cells were then sorted and processed as described in Methods to generate a library of barcoded Ig sequences that was, in turn, curated to analyze the CTD-specific Bmem repertoire, as well as the total nonspecific (CTD^–^) repertoire (Figure 1D). The full gating strategy for sorting CTD^+^ and CTD^–^ Bmem cells and the specificity of CTD binding to B cell antigen receptors (BCR) is depicted in Supplemental Figures 1 and 2.

In addition to flow cytometry, we used a second method to detect CTD^+^ IgG^+^ Bmem cells, following polyclonal stimulation in vitro. PBMCs isolated from subjects 1008, 1009, and 1013 and 3 other subjects, as well as 4 healthy controls, were cultured with polyclonal stimuli to drive differentiation of Bmem cells into new IgG-secreting plasmablasts, which could then be detected by ELISPOT (Supplemental Figure 2). The subjects and controls had a similar frequency of total Bmem cell–derived IgG–secreting cells. None of the controls had CTD-specific IgG–secreting cells. Consistent with previous studies, only 1 of the 6 subjects (subject 1008) showed a clearly positive result, with detectable CTD-specific IgG–secreting cells (33, 54). This shows that CTD^+^ Bmem cells had a poor capacity to differentiate into new plasmablasts following polyclonal stimulation in vitro.

### Lower frequency of class switch in CTD^+^ Bmem cells than in CTD^–^ Bmem cells.

**Figure 2:**
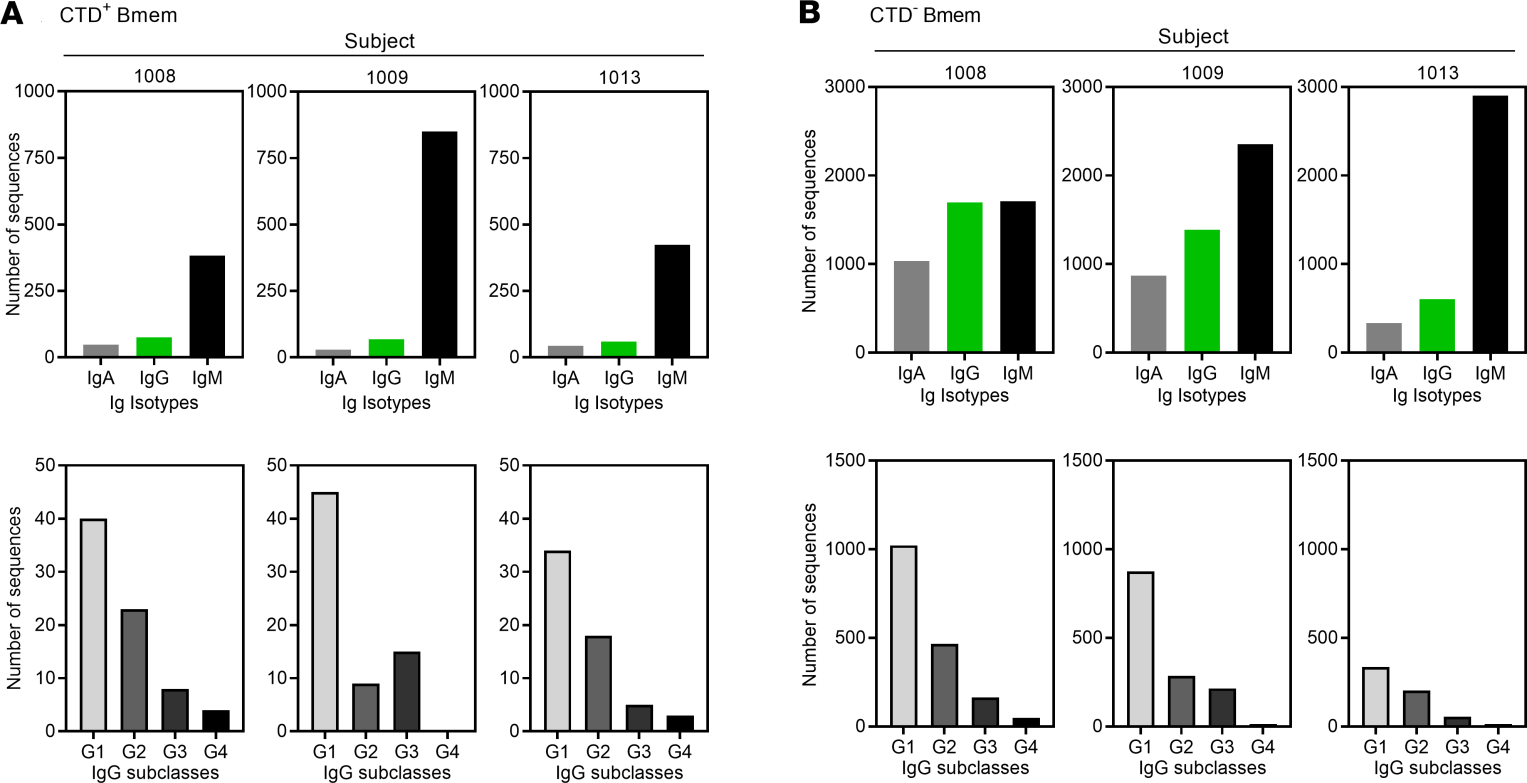
Antibody isotype and subclass distribution in Bmem cells. (**A**) Sequences from CTD^+^ Bmem cells were analyzed for isotype (top row) and IgG subclass distribution (bottom row). *Y* axes denote the number of V(D)J sequences analyzed, representative of the total CTD^+^ Bmem cells analyzed. (**B**) Sequences from CTD^–^ Bmem cells were analyzed as in **A**.

Ig/Ab gene sequences from CTD^+^ and CTD^–^ Bmem cells from subjects 1008, 1009, and 1013 were analyzed, and the heavy chains were grouped according to Ab class and IgG subclass (Figure 2). A lower proportion of the CTD^+^ Bmem cell–encoded Abs were class-switched (to IgA or IgG) as compared with the CTD^–^ Bmem cells. For subjects 1008, 1009, and 1013, the percentages of CTD^+^ Bmem cells that were class-switched to IgG were 14.8%, 7.3%, and 11.4%, respectively (Figure 2A). For the CTD^–^ Bmem cells, 38.2%, 30.1%, and 15.8% of sequences demonstrated class switch, respectively (Figure 2B). The higher ratio of IgM/IgG expression by CTD^+^ Bmem cells became apparent late in the present study. PBMCs were therefore obtained from an additional subject (subject 1018) and cultured with polyclonal stimuli to drive differentiation of Bmem cells and detect CTD-specific IgG as well as IgM-secreting cells by ELISPOT (Supplemental Figure 3). In those analyses, CTD^+^ Bmem cells were dominated by IgM rather than IgG. In contrast, total Bmem cells (representing the CTD^+^ and CTD^–^ populations) had large frequencies of IgM- as well as IgG-secreting cells.

The IgG subclass distribution was analyzed for CTD^+^ and CTD^–^ Bmem cells (Figure 2, A and B). The relative abundance of each IgG subclass was IgG1 > IgG2 > IgG3 > IgG4 in the CTD^+^ and the CTD^–^ Bmem cell compartments. For each subject, there was no discernable difference in the relative numbers of each IgG subclass between the CTD^+^ and the CTD^–^ populations. These data, therefore, suggest an impediment to class switch among CTD^+^ Bmem cells as compared with Bmem cells of other specificities. However, the conditions determining which subclasses are produced appear to be unaltered between CTD^+^ and CTD^–^ Bmem cells.

### Similar Ig heavy and light chain variable gene usage in CTD^+^ and CTD^–^ Bmem.

**Figure 3:**
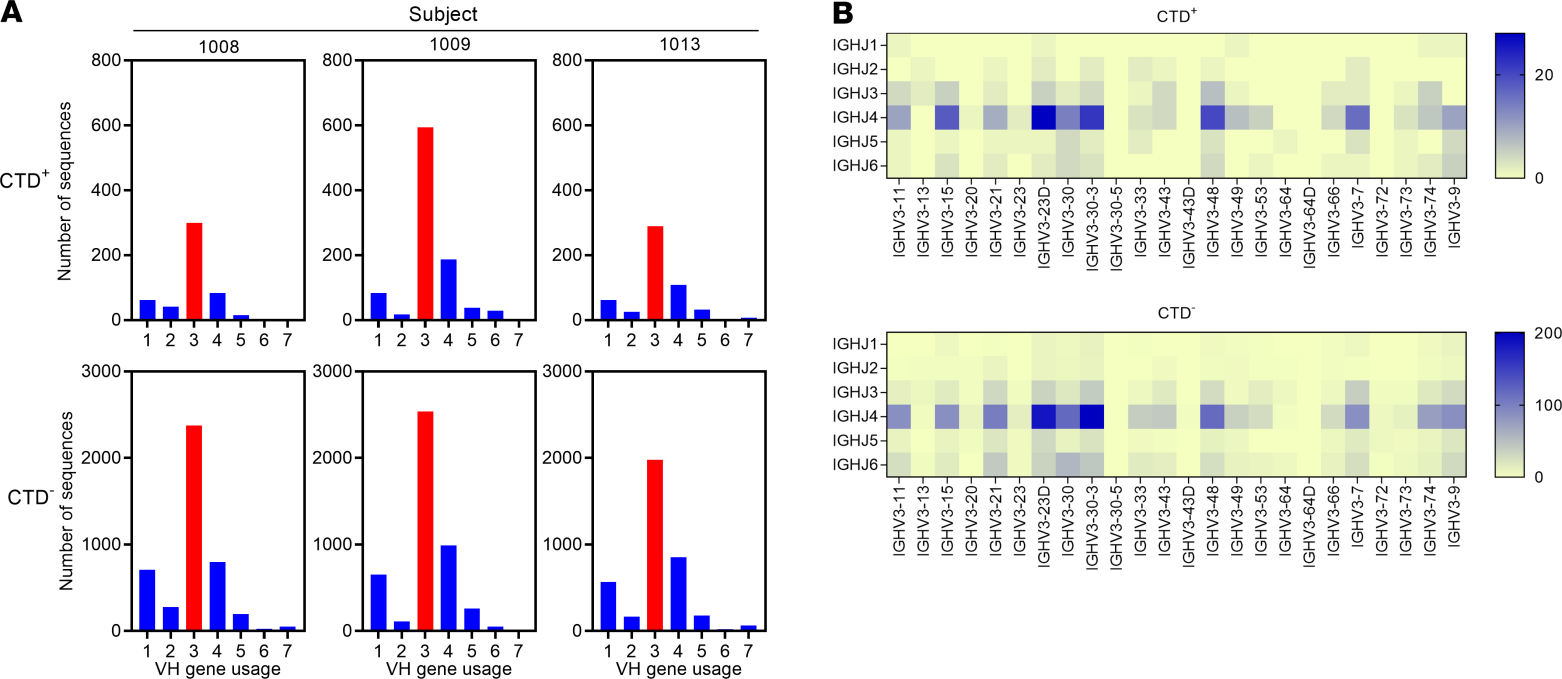
V gene usage in the Ig heavy chain. (**A**) Depicts the V gene usage of CTD^+^ (top row) and CTD^–^ (bottom row) Bmem cells from each subject. IgM, IgG, and IgA sequences were included in the analysis. (**B**) Heatmaps depict the heavy chain V-J gene recombination pairs within the VH3 gene family. Sequences from the CTD^+^ Bmem cells (top panel) and from CTD^–^ Bmem cells (bottom panel) are shown. Data shown are from subject 1008. The color scale indicates the frequency of occurrence of each VH3-J pair.

**Figure 4:**
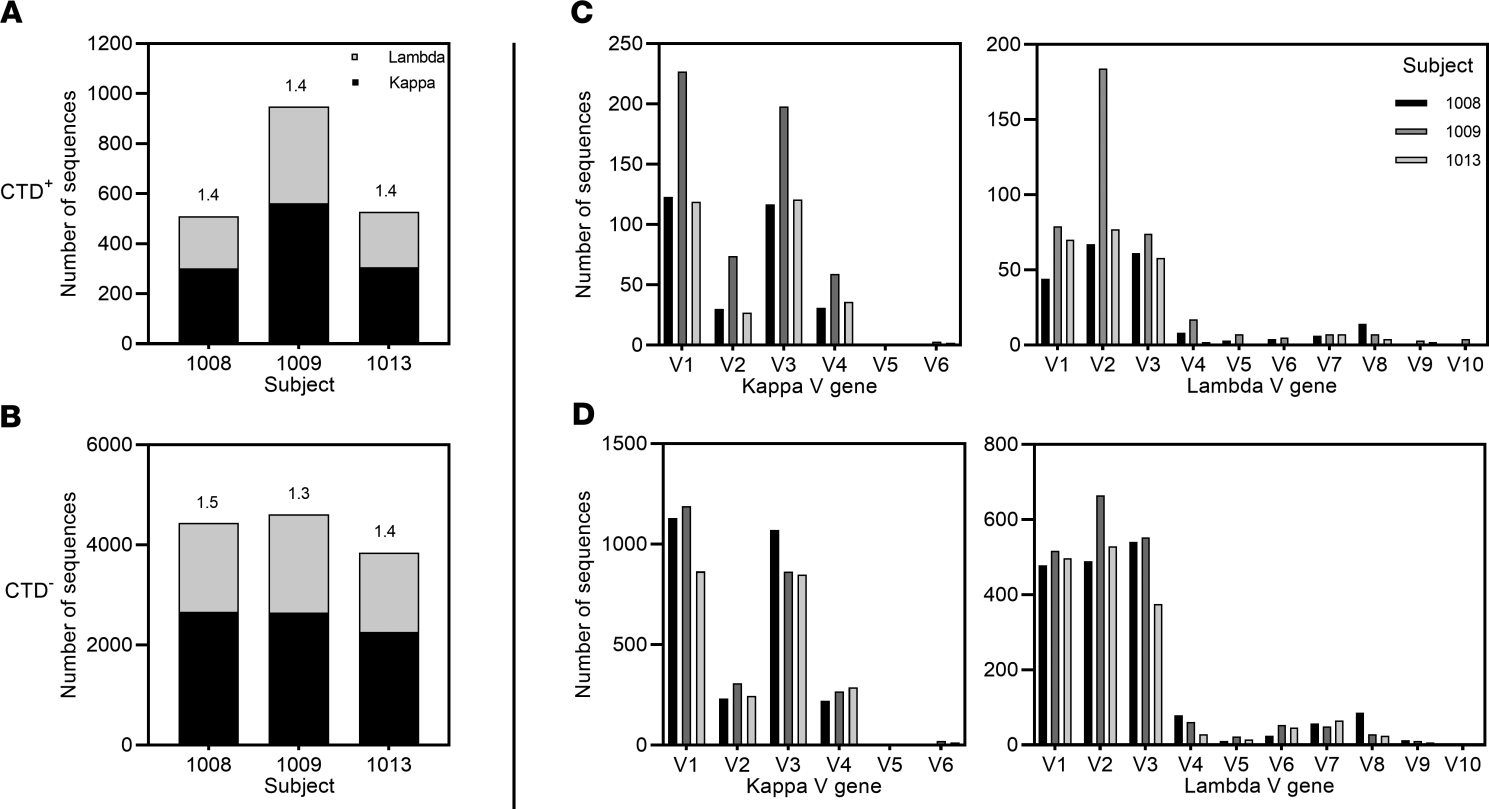
Light chain distribution and their V gene usage in CTD^+^ and CTD^–^ Bmem cells. (**A**) Depicts the number of CTD^+^ Bmem cells expressing a κ or a λ light chain. Numbers above bars denote the κ/λ ratio. (**B**) As in **A**, but depicts κ/λ ratio for CTD^–^ Bmem cells. (**C**) The frequency and distribution of V gene usage by the κ (left) and λ (right) chains by CTD^+^ Bmem is shown. (**D**) As in **C**, but depicts light chain V gene usage by the CTD^–^ Bmem cells.

Repertoire analysis of Bmem cells following specific infections have revealed overrepresentation of select Ig heavy chain variable (IGHV) gene families (58). To understand if *C*. *difficile* skews the IGHV usage in the Bmem cell repertoire, we analyzed the heavy and light chain variable (V) and joining (J) gene usage for CTD^+^ and CTD^–^ Bmem cells. For the CTD^+^ and CTD^–^ Bmem cells of subjects 1008, 1009, and 1013 (Figure 3A), the IGHV3 gene family, and specifically the IGHV3–23 gene was the most frequently used. A detailed analysis of the V-J gene usage within the IGHV3 gene family confirmed that IGHJ4 was the most prevalent IGHJ gene used by CTD^+^ and CTD^–^ Bmem cells (Figures 3B and Supplemental Figure 4, A and B). For the light chain, it was observed that κ was most frequently used, and the typical ratio of κ to λ usage (59) was evident (Figure 4, A and B). Genes V1 and V3 for κ and V1, V2, and V3 for λ were the predominant light chain V genes observed in the study (Figure 4, C and D). Overall, the V gene usage of the CTD^+^ Bmem cells was similar to that observed in CTD^–^ Bmem cells. Furthermore, the IGHV gene usage was similar in subjects 1008, 1009, and 1013.

### Somatic hypermutation in class-switched CTD^+^ Bmem.

**Figure 5:**
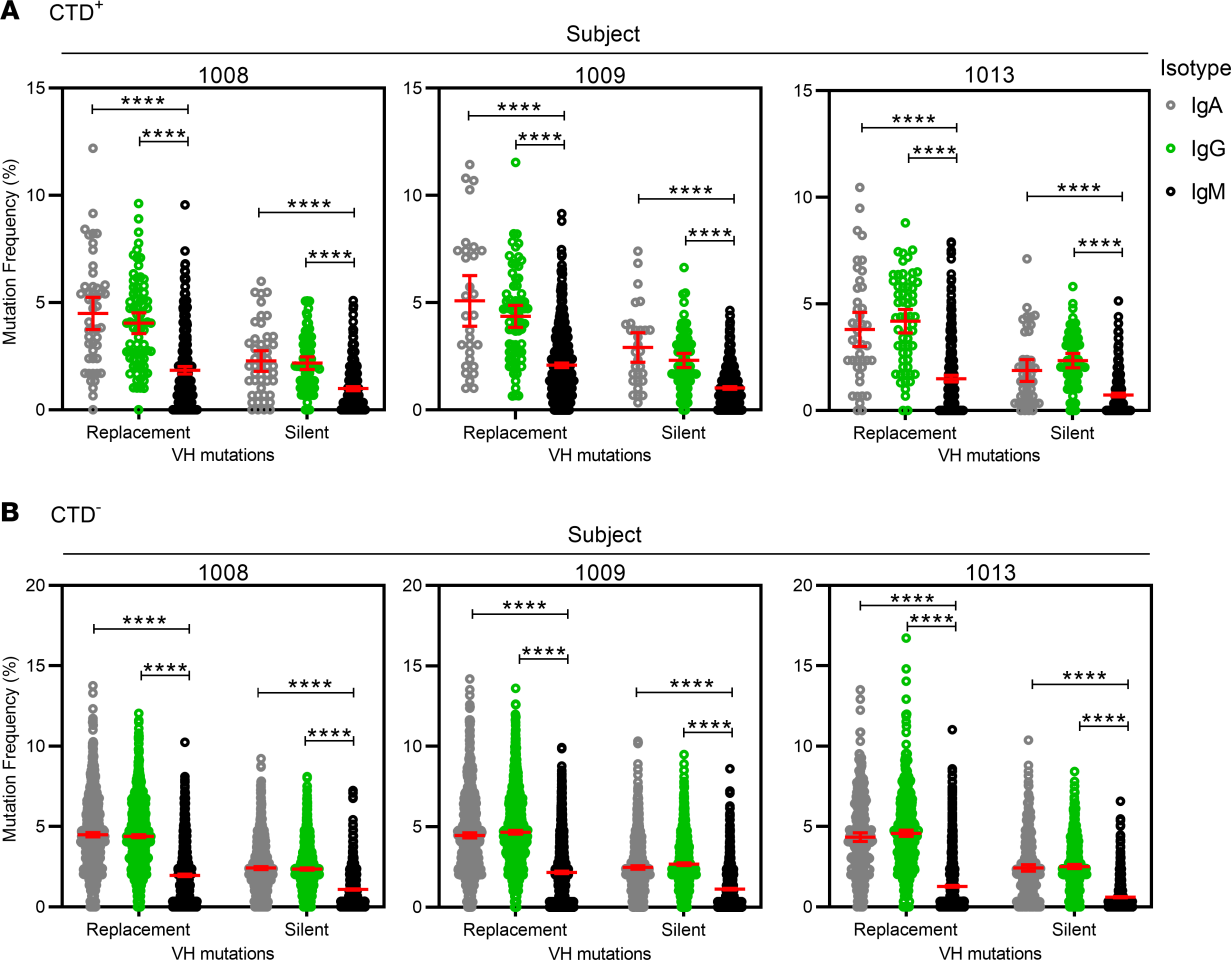
Somatic hypermutation in CTD^+^ and CTD^–^ Bmem cells. (**A**) Depicts the percent nucleotide mutations as compared with germline. Replacement mutations resulting in an amino acid change and silent mutations resulting in no amino acid change in the heavy chain V regions of IgA, IgG, and IgM sequences from CTD^+^ Bmem cells are presented. (**B**) Is as in **A**, but depicts CTD^–^ Bmem cells. A Kruskal-Wallis test with Dunn’s post hoc test correction was used to determine statistical significance in differences between mutation frequencies observed in each Ab isotype (****P < 0.0001).

Mutated V(D)J regions are a hallmark of antigen-experienced B cells. While the recombination of V, D, and J genes create a large repertoire of B cell receptors, the somatic mutations in V regions add further specificity and allow for affinity maturation. In the present study, the IGHV sequences from subjects 1008, 1009, and 1013 were compared with germline sequences to measure the frequency of somatic hypermutation in the V region (Figure 5). As expected, the class-switched sequences (IgA and IgG) from both CTD^+^ and CTD^–^ Bmem had higher frequencies of mutations than the IgM^+^ sequences. This was the case for replacement mutations that caused amino acid changes and silent mutations that did not cause amino acid changes. However, IgM sequences also had replacement mutations. Furthermore, the complementarity-determining regions (CDRs) of the class-switched sequences from the CTD^+^ and CTD^–^ Bmem cells were more frequently mutated than the framework regions (FR) (data not shown). CDRs accumulate more replacement mutations than FRs, allowing CDRs to be more plastic and antigen responsive than the FRs that are tasked with maintaining Ab structure (60, 61). These findings indicated that the CTD^+^ Bmem–encoded Abs were not likely to be deficient in affinity maturation and that some degree of affinity maturation was evident in the IgM compartment.

### Similar characteristics of IgG1-switched CTD^+^ and CTD^–^ Bmem.

**Figure 6:**
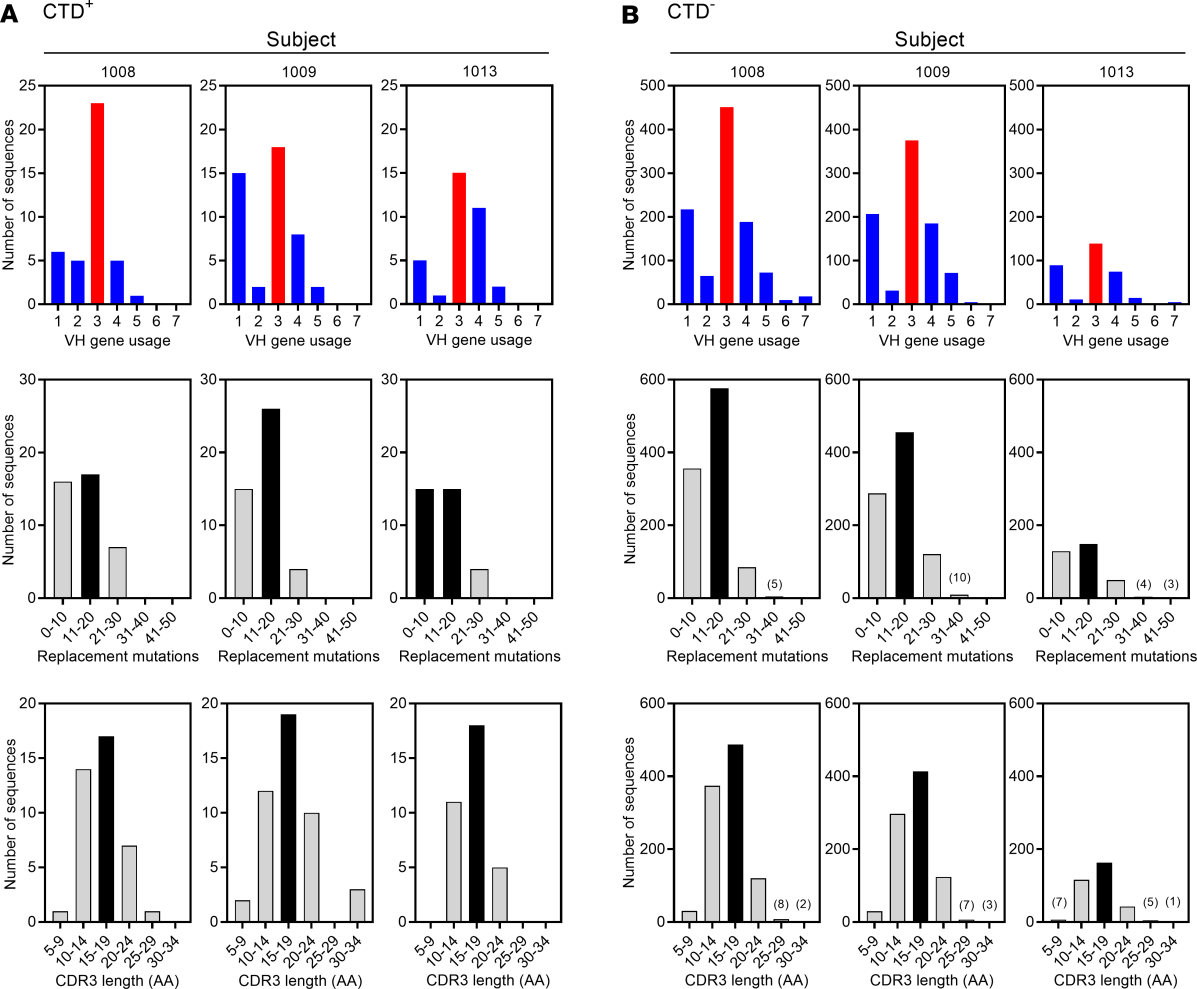
Heavy chain V gene usage, somatic hypermutation and CDR3 length in IgG1^+^ Bmem cells. (**A**) Depicts V gene family usage (top), number of replacement mutations (middle), and CDR3 amino acid (AA) length (bottom) for the CTD^+^ Bmem IgG1 heavy chain sequences. (**B**) As in **A**, but depicts IgG1 heavy chain sequences from CTD^–^ Bmem cells.

Anti–TcdB IgG is the best known correlate of protection against CDI and recurrence (37, 42–45). IgG1 was the predominant IgG subclass observed among IgG^+^CTD^+^ Bmem cells and was analyzed in further detail (Figure 6). For all 3 subjects, the heavy V chain 3 (VH3) was the most used IGHV gene family for IgG1 sequences from CTD^+^ and CTD^–^ Bmem (Figure 6, top). VH1 and VH4 were the next most frequently used genes. Bmem-encoded Abs were characterized by mutated sequences, and the number of IgG1 sequences that had 0–50 replacement mutations in their V region was analyzed (Figure 6, middle). A majority of the sequences had 11–20 replacement mutations in their IGHV regions (consisting of the FR1, FR2, FR3, CDR1, and CDR2 combined). Most of those replacement mutations were in the CDR1 and CDR2 regions (data not shown).

Addition and deletion of nucleotides during V(D)J recombination renders the antigen-binding CDR3 loop heterogeneous (62). Therefore, the range of CDR3 amino acid length in the IgG1 sequences of CTD^+^ and CTD^–^ Bmem cells was determined. More than 50% of the sequences had CDR3 lengths in the 10–20 amino acid range (Figure 6, bottom). Up to 4% had CDR3 lengths of less than 10 amino acids. Only 9%–10% IgG1 sequences from the CTD^–^ subset of all 3 individuals had CDR3 lengths of > 20 amino acids. Sequences with CDR3 lengths of > 20 amino acids in the CTD^+^ subset had frequencies of 15% in subject 1008, 21% in 1009, and 9% in 1013. The CTD^+^ IgG1 sequences in the present study, therefore, displayed features characteristic of an antigen-experienced repertoire.

### Clonal expansion of CTD-specific IgM antibody.

**Figure 7:**
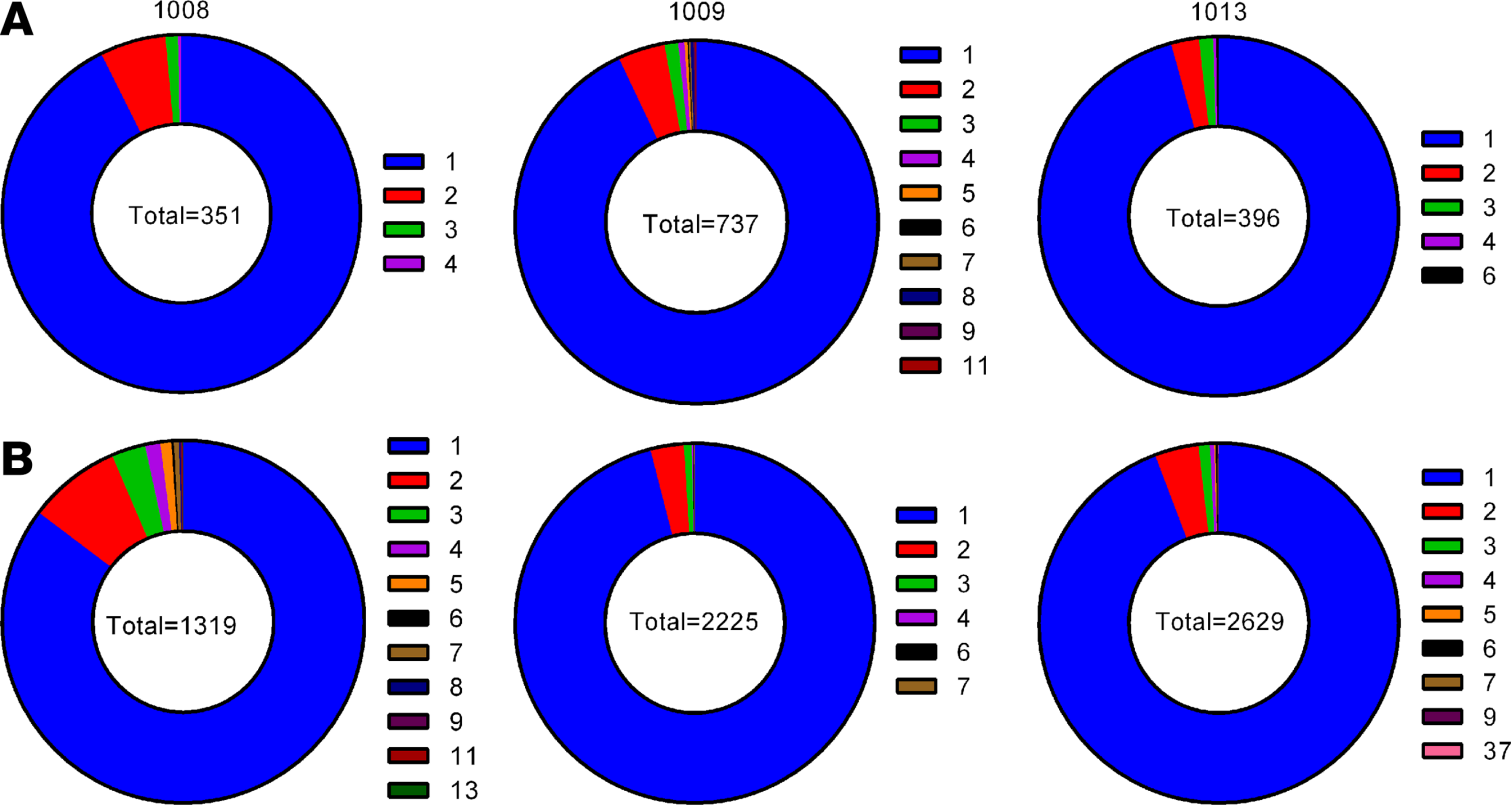
Clonal expansion in IgM^+^ Bmem cells. (**A** and **B**) Graphs depict the clonal expansion in IgM^+^ sequences from CTD^+^ (**A**) and CTD^–^ (**B**) Bmem cells. The number in the center of each chart denotes the number of sequences analyzed. The numbers in the legends to the right of each chart indicate the size of a given clone. The shaded areas represent the frequency with which clones of each size appeared within the total sample.

The human Bmem cell compartment is characterized by a high degree of clonal diversity (63). The clonal families observed in class-switched and non–class-switched CTD^+^ and CTD^–^ Bmem cells from subjects 1008, 1009, and 1013 were therefore analyzed. A majority of the CTD^+^ and CTD^–^ Bmem populations were polyclonal, containing sequences unique within the cell sample analyzed (Figure 7). IgM^+^ Bmem cells demonstrated several expanded clones. One CTD^+^ Bmem clone from subject 1009 had 11 members, while a CTD^–^ Bmem clone from subject 1013 had 37 members (Figure 7).

Class-switched Bmem cells largely consisted of unique clones. All IgA^+^ clones were unique except for 1 clone with 2 members (in CTD^+^ Bmem of subjects 1008 and 1009). The IgG^+^ clones in CTD^+^ Bmem from subjects 1008 and 1009 were unique. Subject 1013 had 1 IgA^+^ clone with 4 members and 1 IgG^+^ clone with 2 members. Furthermore, clones identified from each subject were unique to that individual.

The CTD^–^ Bmem cells from the 3 subjects demonstrated limited clonal expansion in the IgA^+^ Bmem cell compartment (ranging from 38 clones with 2 members to 1 clone with 7 members). Similarly, in the IgG^+^ Bmem cell compartment, there was a range of 52 clones with 2 members to 1 clone with 4 members (data not shown).

While there was evidence of clonal expansion in CTD^+^ Bmem cells, the expansion was largely restricted to a few IgM^+^ clones, suggesting that CDI may result in a Bmem cell compartment composed of several unique clones.

### Validation of sequencing and analytical strategy using Bmem cells from a healthy control.

Validation of the barcoding, sequencing and analytical methods used in this study was provided by analyzing total Bmem cells from a healthy control (subject 1007) (Supplemental Figures 5 and 6). Enriched B cells were harvested and CD3^–^CD19^+^CD20^+^CD27^+^CD38^–^IgM^+^ and CD3^–^CD19^+^CD20^+^CD27^+^CD38^–^IgM^–^ Bmem cells were sorted for heavy chain V; diversity (D); and J, light chain V, and J repertoire analysis (Supplemental Figure 5A). The healthy IgG subclass distribution (Supplemental Figure 5B), the V region usage (Supplemental Figure 5C), and the mutation frequency in IgA, IgG, and IgM (Supplemental Figure 5D) were as expected. Further analysis of the mutation frequency in IgG1^+^ Bmem cells (Supplemental Figure 6A), the CDR3 length (Supplemental Figure 6B), and the clonality of the IgM, IgG, and IgA compartments (Supplemental Figure 6C) were also as expected. These features were characteristic of a Bmem cell compartment and served to provide validation for the methods described in analysis of the Bmem repertoire from experimental subjects.

### Characteristics and functional capacity of fully human full-length anti-CTD Abs.

**Figure 8:**
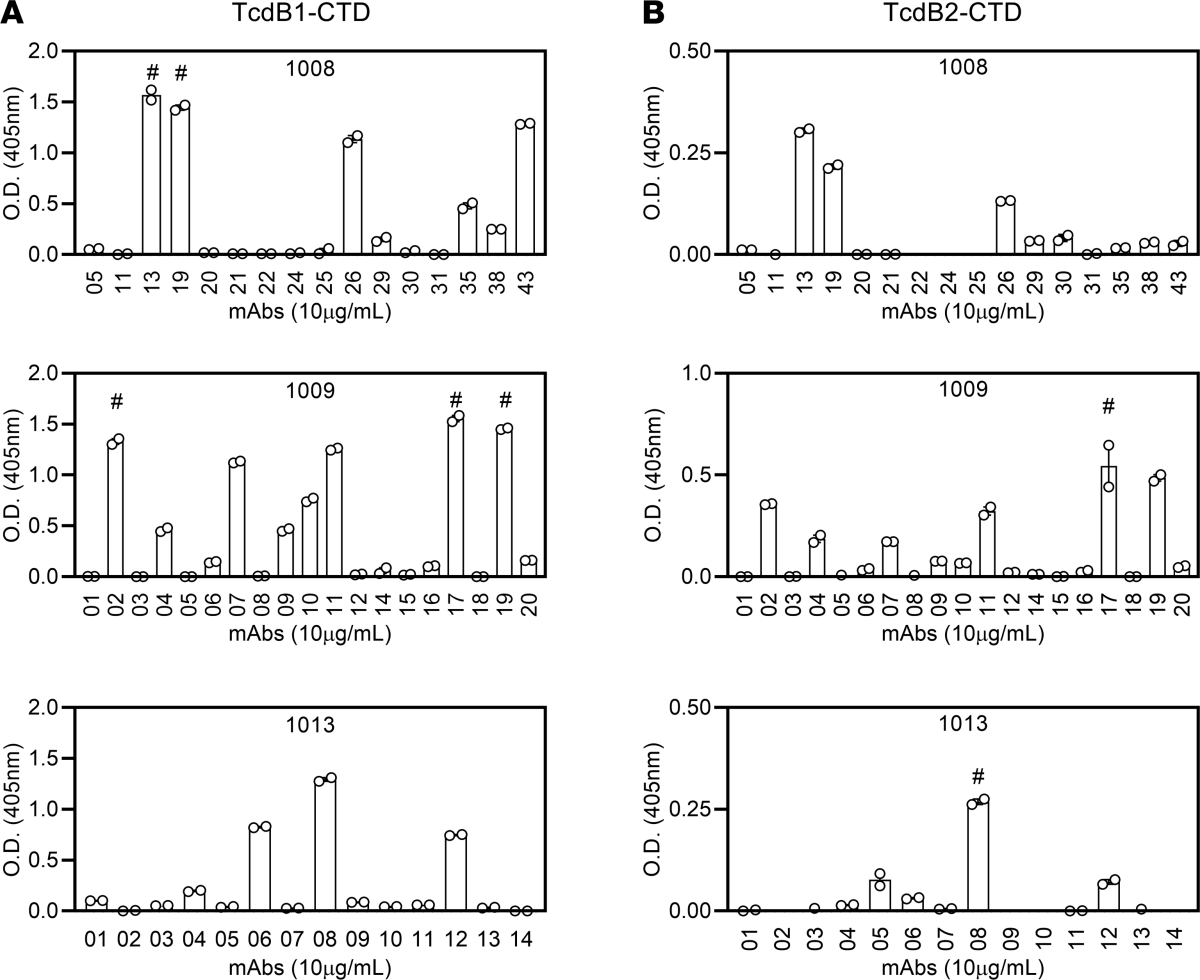
Bmem-encoded mAbs bind the CTD region of TcdB1 and TcdB2. Select IgG1 heavy and light chain sequences from CTD^+^ Bmem cells were cloned and transfected into HEK293 cells to generate physiologically paired, full-length human mAbs. (**A**) TcdB1-CTD binding by mAbs from subjects 1008 (top), 1009 (middle), and 1013 (bottom) were tested by ELISA. The symbol (#) above a bar indicates the mAbs that were analyzed for CTD-binding affinity. Duplicates from a single experiment are presented and are representative of at least 2 independent determinations. (**B**) As in **A**, except TcdB2-CTD binding was analyzed. The data are represented by mean ± SEM.

**Figure 9:**
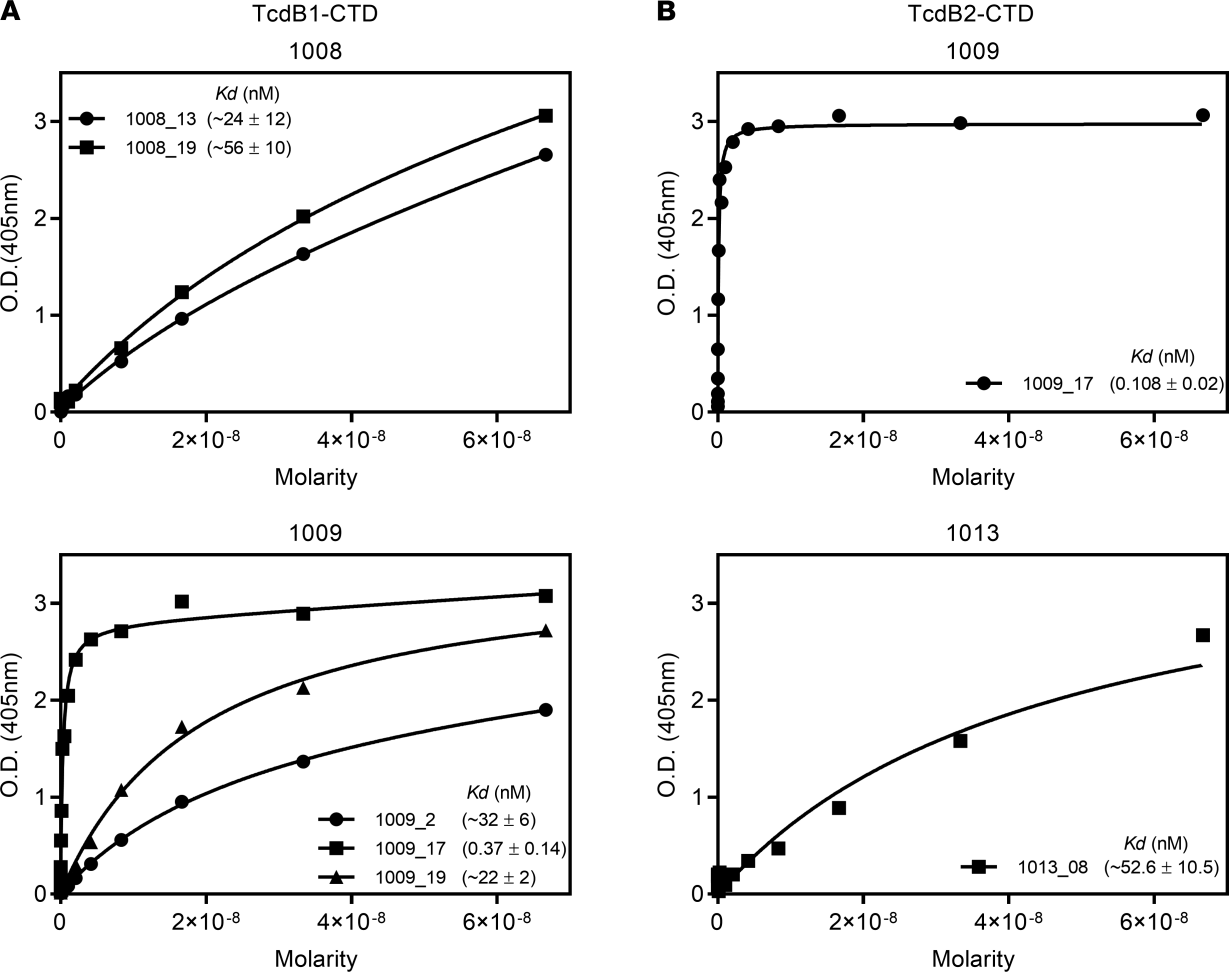
Bmem-encoded mAbs bind CTD with low to moderate affinity. (**A**) Select mAbs from subjects 1008 (top) and 1009 (bottom) were subjected to a 16-point dilution curve to calculate their binding constants (*K_D_*) to analyze their binding affinity to TcdB1-CTD. The *K_D_* values are as indicated. (**B**) As in **A**, except the binding affinity to TcdB2-CTD for select mAbs from subjects 1009 and 1013 was analyzed. Each experiment was performed at least 3 times with the same results.

The best known correlate of protection against primary and recurrent CDI is TcdB-neutralizing IgG of potentially high affinity (37, 42–45, 50, 51). Experiments were therefore performed to test the antigen binding and functional activity of the IgG1 sequences that were observed in the CTD^+^ Bmem cell compartment. Several mAbs were generated from the IgG1 sequences obtained from subjects 1008, 1009, and 1013. The sequences were selected to represent the VH gene usage, range of CDR3 lengths, and numbers of mutations in the CTD^+^IgG1^+^ Bmem sequences (Supplemental Table 2). The mAbs were tested for antigen binding and affinity (Figure 8 and Figure 9). Of the mAbs generated, 24 of 49 bound by ELISA to CTD from TcdB1 and 22 of 49 mAbs bound to CTD from TcdB2 (Figure 8). One of the 6 mAbs tested (mAb 1009_17) bound both TcdB1-CTD and TcdB2-CTD with a less-than-nanomolar affinity (Figure 9). mAb 1009_17 bound TcdB1-CTD with dissociation constants (*K_D_*) = 0.37 ± 0.14 nM and TcdB2-CTD with *K_D_* = 0.108 ± 0.02 nM. Saturated binding could not be achieved for all mAbs, consistent with low to moderate affinity, and are designated in the figure as approximate (~) values.

**Figure 10:**
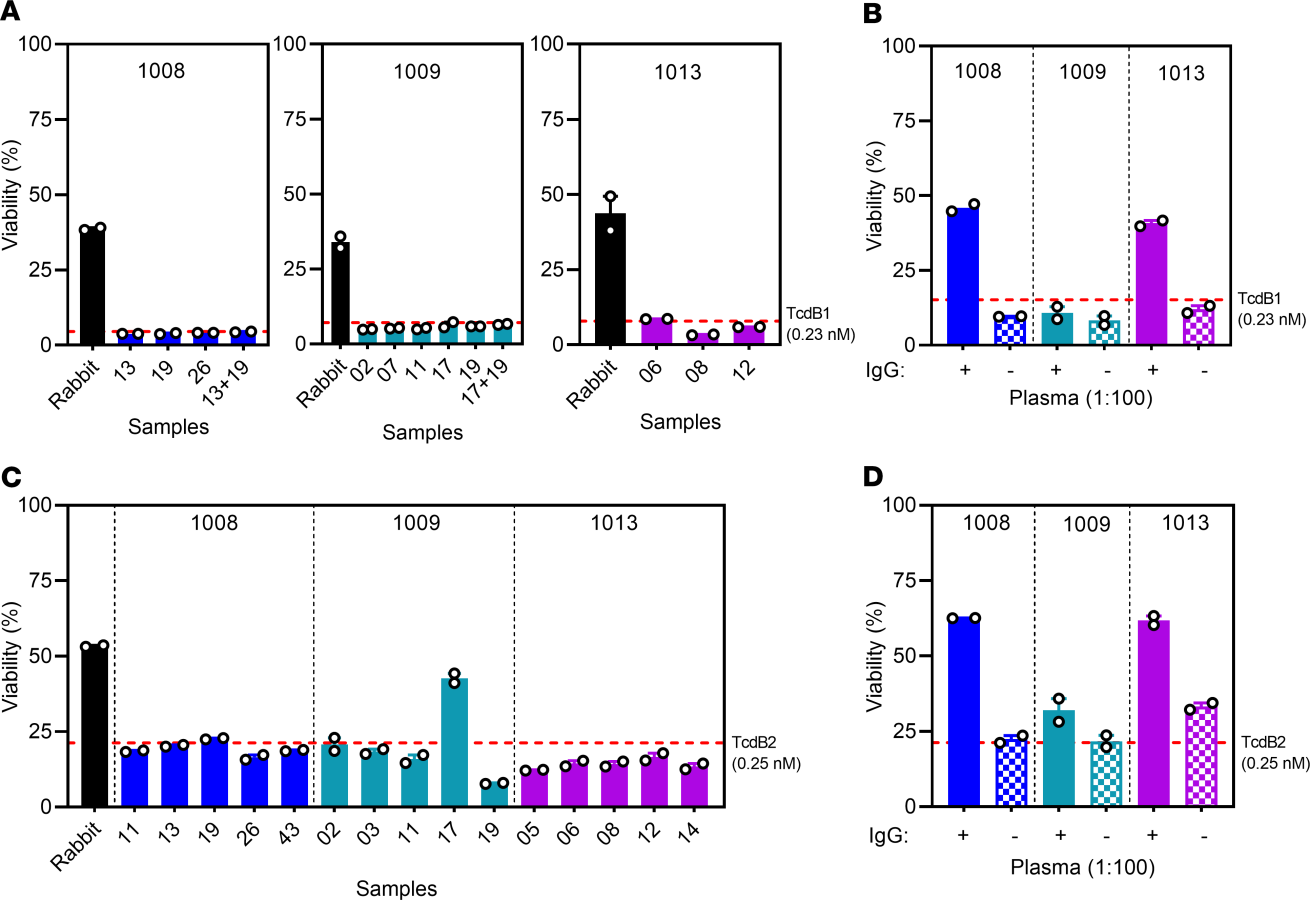
CTD-specific Bmem encode nonneutralizing antibodies. (**A**) Serum from CTD-immunized rabbit diluted to 1:100 and mAbs from subjects 1008, 1009, and 1013 at a final concentration of 10 μg/mL mixed with media containing 0.23 nM TcdB1 were added to CHO cells to assess cell viability. (**B**) IgG from plasma of subjects 1008, 1009, and 1013 was depleted and tested in vitro for toxin neutralization capacity. The plasma with and without IgG were diluted 1:100 in media with 0.23 nM TcdB1. (**C** and **D**) As in **A** and **B**, respectively, except samples were mixed with media containing 0.25 nM TcdB2 and added to CHO cells to assess cell viability. Red dotted line represents CHO cell viability in the presence of media containing toxin alone. Duplicates from a single experiment are presented and are representative of at least 2 independent determinations. The data are represented by mean ± SEM.

The Bmem-encoded mAbs from the 3 subjects were then tested for their ability to neutralize in vitro intoxication of CHO cells by TcdB1 and TcdB2 (Figure 10). The mAbs from 1008, 1009, and 1013 tested individually or in a variety of combinations were unable to neutralize TcdB1 activity in vitro. However, mAb 1009_17 was able neutralize TcdB2 activity in vitro, resulting in ~60% CHO cell viability (Figure 10, A and C).

CTD^+^IgG1^+^ Bmem cells lacked clonal expansion and encoded mAbs with limited toxin-neutralizing capacity. We therefore selected sequences from the clonally expanded IgM^+^CTD^+^ Bmem cells of subjects 1008, 1009, and 1013 (Supplemental Table 2). The IgM V(D)J regions were expressed as IgG1 mAbs to control for differences between IgM and IgG1 constant regions. The IgM-V(D)J/IgG1 mAbs were then tested for antigen binding and toxin neutralization activity in vitro (Supplemental Figure 7). Only 2 of 8 mAbs generated showed binding to TcdB1-CTD by ELISA. None of the mAbs demonstrated TcdB1 neutralization in vitro.

Plasma obtained from subjects 1008 and 1013, but not 1009, was able to neutralize both TcdB1 and TcdB2 in vitro. To confirm that the IgG in the plasma was primarily responsible for the neutralization, we depleted IgG from the plasma of subjects 1008 and 1013 and observed that the samples lost their capacity to neutralize toxin in vitro (Figure 10, B and D). CDI therefore resulted in generation of a toxin-specific plasma cell–derived polyclonal Ab pool containing sufficient amounts of neutralizing Ab for in vitro toxin neutralization.

## Discussion

Typically, the host immune system establishes “memory” to a primary infection, resulting in a rapid and effective response upon reexposure to a pathogen. A recent study from our laboratory revealed that an initial infection of mice with *C*. *difficile* spores did not induce a CTD/TcdB-specific Bmem response or protect from pathology associated with repeat infection (64). This is consistent with another study from our laboratory where human subjects with a history of CDI demonstrated poor differentiation of CTD-specific Bmem cells into new plasmablasts following polyclonal stimulation (33). Recurrent CDI could therefore result from an inadequate Bmem cell response. Studies with human subjects have shown that neutralizing serum IgG specific for TcdB is associated with protection against primary and recurrent infections (42, 43). However, the human TcdB-specific Bmem repertoire and whether it encodes TcdB-neutralizing Ab has not been evaluated. This is the first study, to the best of our knowledge, that determined the repertoire and function of CTD^+^ Bmem from individuals with a self-reported history of CDI.

Antigen-specific Bmem cells occur in low frequencies in human PBMCs. We therefore isolated TcdB1-CTD^+^ and -CTD^–^ Bmem cells, single-cell barcoded them, and then examined their Ig genes by deep sequencing. Surprisingly, the CTD^+^ Bmem cells had a much lower proportion of class-switched Abs than the CTD^–^ Bmem cell population. These IgM^+^ sequences from the CTD^+^ Bmem cells had undergone somatic hypermutation, as evidenced by changes compared with germline sequences. The presence of mutations in the IgM sequences in conjunction with our cell-sorting strategy, which excluded CD38^+^ cells, confirmed that these were not natural Ab-secreting cells (65). We observed a much higher frequency of IgM^+^ rather than IgG^+^CTD^+^ Bmem cells in our ELISPOT assays, as well. These findings are consistent with our observation of limited class switch and IgM dominance in our recently published study using a mouse model of *C*. *difficile* recurrence (64).

IgM^+^ Bmem cells have been observed in human PBMCs in response to T-independent antigens such as polysaccharides (66) and malaria infection (67). To the best of our knowledge, this is the first report demonstrating a predominantly IgM^+^ Bmem cell compartment following CDI. Several of the CTD^+^ IgM sequences were clonally related, forming clones with up to 11 members. Recent studies have described IgM that can neutralize Chikungunya virus early in infection (68), that can block HIV-transmission in cervico-vaginal tissues (69), and that can afford protection against Influenza (70). However, we observed that the CTD^+^IgM^+^ Bmem cell–encoded Ab did not neutralize TcdB1-CTD. We did not anticipate Bmem-encoded CTD–specific IgM to play a role in neutralizing toxin, since there are several studies that have demonstrated IgG to be the best correlate of protection to CDI (37, 42–45). Furthermore, the IgG-depleted plasma samples in our study were unable to neutralize toxin in vitro, emphasizing the role of IgG in preventing recurrent CDI.

Analysis of IGHV gene usage in adults with rotavirus-experienced B cells show bias in their use of the VH1 and VH4 genes (71). VH analysis in B cells from individuals with autoimmune disease have demonstrated VH4 and VH5 biases in systemic lupus erythematosus (SLE) and VH3 bias in myasthenia gravis (72). To understand if CDI resulted in a biased VH repertoire, we analyzed the VH gene usage of CTD^+^ and CTD^–^ Bmem cells and found no differences between them. Consistent with previous studies involving healthy individuals, we observed that the V3 and J4 gene families dominated the heavy chain repertoire (73–75). The IGHV regions from class-switched CTD^+^ Bmem cells, from CTD^+^IgM^+^ Bmem cells, and from CTD^–^ Bmem cells had undergone somatic hypermutation. This demonstrates that the affinity maturation process in patients was intact.

The ratio of replacement to silent mutations in the CDR1 and -2 regions of several class-switched sequences from CTD^+^ Bmem cells was > 2.9, suggesting antigenic selection of those clones (76). Although IgG only accounted for about 10% of the CTD^+^ Bmem cells, the subclass distribution was similar to that observed for the CTD^–^ Bmem cells, with IgG1 accounting for a majority of the IgG^+^ sequences. As is typical for class-switched Bmem cells, the IgG1 sequences had CDR3 lengths of up to 20 amino acids and an increased VH1 usage as compared with memory IgM sequences (77). Overall, these findings suggested that the CTD^+^ Bmem cells encoded a repertoire that was theoretically capable of neutralizing TcdB. For this reason, several IgG1 mAbs were generated from the sequences obtained from each subject to test their binding specificity to and their affinity for CTD from TcdB1 and TcdB2. We also tested the ability of these IgG1 mAbs to neutralize TcdB1 and TcdB2 in an in vitro CHO cell viability assay. While almost 50% of the 49 IgG1 mAbs generated bound CTD by ELISA, only 1 mAb (mAb 1009_17) bound with high affinity (*K_D_* = 0.108 ± 0.02 nM) and neutralized TcdB2 to prevent CHO cell killing in vitro. This supports the possibility that CTD-specific Bmem cells encode mostly low-affinity Abs incapable of toxin neutralization. Bezlotoxumab, a TcdB-targeting human mAb FDA-approved only for prevention of recurrent CDI (78, 79), binds high-affinity sites in the full-length TcdB1 with a *K_D_* of 19 ± 5 pM (49). This *K_D_* is 19-fold greater than the *K_D_* with which the highest-affinity mAb expressed in this study binds TcdB1 (mAb 1009_17; *K_D_* = 0.37 ± 0.14 nM). While the CTD binding site for bezlotoxumab has been characterized (49), the specific binding sites for the mAbs generated in this study remain to be identified.

It should be noted that although CTD-binding and toxin-neutralizing Bmem cell–encoded mAbs were generated for this study, the question remains as to whether these Bmem cells have the capacity to differentiate in response to CDI and express Abs in vivo. This is attested by the inability of plasma from subject 1009 to neutralize toxin in vitro, although their Bmem cell compartment encoded a toxin-neutralizing mAb. In our recent murine studies, CDI resulted in a poor expansion of PD-1^hi^CXCR5^+^ follicular helper T cells (Tfh) (64). This lack of T cell help could limit Bmem cell expansion and differentiation into Ab-secreting plasma cells. The role of Tfh in this study could not be explored, since the subject samples were obtained several months after infection.

It is also possible that CTD-specific Bmem cells encode low-affinity Abs incapable of toxin neutralization. This is supported by the mostly low- to moderate-affinity mAbs produced from the CTD-specific Bmem cell pool in the present study and the observation that the only mAb (mAb 1009_17) that bound TcdB2-CTD with high affinity also successfully neutralized TcdB2 in an in vitro viability assay.

Differences in disease severity could also influence the Bmem response. Although subjects 1008, 1009, and 1013 self-reported a single infection in the 5 years before recruitment into this study, they all reported being symptomatic. Subjects 1008 and 1013 reported receiving medical treatment, and only 1013 reported being hospitalized due to CDI. Due to the lack of complete medical history, we cannot comment on the influence of disease severity on the humoral response in our cohort. An alternative hypothesis is that Bmem-encoding neutralizing Ab may only develop after several recurrent infections, at which point the individual may not benefit from those Abs due to the extensive gut damage from previous infections. Comparing the Bmem cell repertoires in individuals following recurrent and nonrecurrent infections may allow testing of this hypothesis, but the high mortality rate in individuals with recurrent disease may preclude a larger study.

It is very likely the study participants could have been infected with a *C*. *difficile* strain other than the historical VPI-10463 strain; therefore, their Bmem-encoded Abs did not neutralize TcdB1. While Abs produced in response to TcdB2 from the hypervirulent NAP1/BI/027 strain cross-react with TcdB1 from the historical strain, the cross-neutralization between strains is limited (28, 33, 80). This was confirmed in our analysis where 1 mAb from subject 1009 bound CTD from both TcdB1 and TcdB2 but only neutralized TcdB2 in vitro. In this study, we tested the CTD-specific Bmem response, but the response to TcdB (whole toxin) could arguably be broader. However, we observed that the plasma anti–TcdB1-CTD IgG response was very similar to the anti–TcdB1 IgG response, and the same plasma samples had background level reactivity to a recombinant TcdB1 glucosyltransferase domain (data not shown). These findings, combined with previously published literature measuring the antigenicity and neutralizing capacity of CTD (33, 55, 56), indicate a predominantly CTD-specific Bmem repertoire.

The lack of TcdB1 neutralization by the Bmem-encoded mAbs appeared to contrast with that of plasma samples from subjects 1008 and 1013, which neutralized toxin in an IgG-dependent manner. A discrepancy between plasma antibody titers and circulating Bmem cell frequency and function has been documented for *C*. *difficile* and other bacterial toxins and has been proposed as a feature of the host humoral response to bacterial toxins (54, 81). Although this study was not designed to investigate differences between Bmem and plasma cell–derived Abs, there could be several explanations for this observation. Arguably, the simplest explanation is that only a small proportion of plasma cell–derived Abs are toxin neutralizing, but sufficient neutralizing Abs had accumulated in the sera to be functional in the in vitro assay. Similarly, very few Bmem cells may encode neutralizing Ab, explaining why only 1 mAb of 49 was neutralizing. The TcdB-neutralizing Ab observed in plasma samples is likely composed of numerous-fine specificities targeting several epitopes on the toxin, allowing more efficient toxin neutralization. Plasma from subjects 1008 and 1013 neutralized both TcdB1 and TcdB2 in vitro, possibly alluding to a requirement for combating CDI with Abs of multiple specificities. In this study, it was observed that several cocktails of 3 different Bmem cell–derived anti-CTD mAbs failed to neutralize toxin in vitro.

Finally, a caveat to extrapolating from in vitro toxin neutralization studies is that they may not accurately predict in vivo protection (82, 83). Toxin concentration and availability of neutralizing Abs in the gut lumen, as well as rate of toxin clearance, are likely to influence disease progression, severity, and treatment of CDI (26, 84, 85).

Several vaccine candidates to prevent CDI are in clinical trials, and while they show promise, their efficacy remains to be determined (86). Our study highlights the importance of considering the basic immune response to this infection, as well as the necessity of designing and testing new vaccines. In an animal model, immunization with CTD followed by CDI did not generate a recall response (64), and the animals were protected due to the Ab secreted by plasma cells generated in response to prior immunization. Similarly, this study demonstrated that CDI did not establish an efficient memory response in humans. This implies a requirement for repeated vaccinations to sustain an adequate protective response to future infections.

Further studies comparing Bmem cell repertoires in larger cohorts of individuals with single episodes of CDI or recurrent CDI will allow a deeper understanding of how *C*. *difficile* impacts the host humoral immune response. Comparing the plasma cell–derived Ab repertoire with that of the Bmem-derived Ab repertoire may provide further insights. Finally, the immune response to *C*. *difficile* antigens other than the toxin need to be evaluated.

## Methods

Supplemental Methods are available online with this article.

### Human subject eligibility, recruitment, and selection.

The present study was designed to identify individuals with a history of CDI and determine their Bmem-encoded Ab repertoire. To achieve this goal, blood samples were collected from males and females who either had CDI within the past 5 years (subjects) or had no known history of CDI (controls). Individuals aged 20–85 and asymptomatic at the time of the blood draw were eligible for the study. Individuals with a history of major health issues including autoimmune disease, cancer, cardiovascular disease, or ongoing infectious disease (including HIV) were excluded from the study. Individuals who were presently unhealthy, had been vaccinated within the past month, or weighed less than 110 pounds were also excluded. Subjects included in this study self-reported receiving a laboratory-based diagnosis for CDI and reported no complications from CDI. Subjects 1008 and 1009 self-reported single episodes of CDI at the time of recruitment. Subject 1013 self-reported 2 episodes 3 years apart, with the first episode occurring almost 6 years before recruitment in this study, and was hospitalized due to the second episode. Since the second episode for subject 1013 did not occur within 12 weeks of the first infection, it did not meet the criteria for recurrent CDI (51). Blood samples were analyzed to determine if individuals had a detectable population of TcdB-specific (CTD fragment of TcdB) Bmem cells. Subjects 1008, 1009, and 1013 had demonstrable CTD-specific Bmem cells in peripheral blood as detailed in the Results and were available for a second visit to provide sample for repertoire analysis. Repertoire analysis was therefore performed on CTD^+^ and CTD^–^ Bmem cells from subjects 1008, 1009, and 1013. To validate our isolation, sequencing, and analytical methods, we isolated total Bmem cells from a healthy control (subject 1007) for repertoire analysis. Details for individuals included in this study are presented in Supplemental Table 1.

### Sample procurement, PBMC preparation, and B cell enrichment.

Peripheral venous blood was drawn from healthy subjects into vacuum tubes containing acid citrate dextrose (BD Biosciences). Samples were centrifuged for 15 minutes at 400 *g* at room temperature to collect plasma. Packed blood was mixed with an equal volume of 1 × PBS, layered onto 15 mL of lymphocyte separation medium, known as LSM (Lonza), and centrifuged at 800 *g* (no brake) for 30 minutes at room temperature. PBMCs collected using a pipette were washed with PBS and resuspended in PBS containing 2% v/v FCS. PBMCs were used for initial flow cytometry analysis. The RosetteSep Human B cell enrichment cocktail (Stemcell Technologies) was used for B cell enrichment according to the manufacturer’s instructions. Briefly, whole blood was mixed with RosetteSep cocktail (50 μL/mL of blood sample) and allowed to incubate for 20 minutes at room temperature. The sample was then diluted by adding an equal volume of PBS/2% v/v FCS. After mixing gently, the diluted sample was layered onto LSM and centrifuged at 1200 *g* (no brake) for 20 minutes at room temperature. The enriched B cell population was collected from the top of the LSM layer, washed in PBS, and resuspended in PBS/2% v/v FCS. The enriched B cell samples were used as starting material for Bmem cell labeling and sorting.

### Flow cytometry and cell sorting.

Where indicated, PBMCs or enriched B cells were incubated with flurochrome-conjugated mAbs against cell surface proteins at 4°C for 30 minutes, before washing 3 times with PBS containing 2% FCS. The following anti-human mAbs were used: PE-Alexa Fluor 610 CD19 (clone SJ25C-1), Pacific Orange CD20 (clone HI47) APC–Alexa Fluor 750 CD27 (clone CLB-27/1), and APC-Cy5.5 CD38 (clone HIT2) from Invitrogen; PE-Cy7 CD3 (clone UCHT1) and APC IgG (clone G18-145) from BD Biosciences; and PE-IgM (clone UHB) from SouthernBiotech. Data were collected using a FACSAria III instrument and analyzed using FlowJo_V10 software (BD Biosciences). For Bmem cell isolation, enriched B cells were first selected for size and granularity using FSC/SSC followed by gating for singlets. The CD3^–^CD20^+^CD19^+^CD27^+^CD38^–^ cells were then selected to obtain Bmem cells. CD20^+^CD27^+^CD43^+^CD70^–^CD38^+^CD5^+^ B1 B cells were therefore not collected during sorting (65). Where indicated, cells were counterstained with Alexa 488–CTD to facilitate simultaneous sorting of CTD^+^ and CTD^–^ Bmem cells. B cell receptor blocking was achieved by adding 20 μL goat anti-human IgG, IgM, and IgA (H&L, catalog GWB-A3E324) from GenWay Biotech per 2 × 10^6^ PBMCs. After a 30-minute incubation at 4°C, the samples are washed once with PBS containing 2% FCS before adding Alexa 488–CTD.

### Expression and purification of TcdB and CTD and CTD labeling.

TcdB from strain VPI-10463 and NAP1/BI/027 was purified using established methods (28, 87). The CTD-encoding region of the TcdB gene was codon optimized and cloned into the pET15b plasmid (GenScript) as described previously (33). Briefly, the VPI-10463 and NAP1/BI/027 CTD genes were amplified using primers 5′-GATCATATGCTGTATGTGGGTAACCG-3′ and 5′-AACGGATCCTTATTCGCTAATAACCA-3′, and restriction sites BamHI and NdeI were included for cloning into pET15b. VPI-10463 TcdB is referred to as TcdB1 and NAP1/BI/027 TcdB is referred to as TcdB2. VPI-10463 CTD (TcdB1 amino acids 1651–2366) and NAP1/BI/027 CTD (TcdB2 amino acids 1651–2366) were expressed in *E. coli* BL21 star DE3 (Invitrogen) and purified by Ni2^+^ affinity chromatography (HisTrap; GE Life Sciences). The holotoxin and CTD are expressed and purified using the same method, and the structure of the CTD obtained maintains the same structure as the holotoxin (49). Mouse splenocytes were cultured with the CTD preparation, which failed to cause polyclonal B cell expansion and IgM secretion, as is expected for LPS, thus confirming sufficient LPS removal during purification. An Alexa Fluor 488 protein labeling kit (Invitrogen) was used to label CTD from TcdB1, according to the manufacturer’s instructions.

### Barcoding and library construction.

Sorted CTD^+^ and CTD^–^ Bmem cells were processed using the Chromium Single Cell V(D)J Enrichment Kit for human B cells in conjunction with a Chromium controller. This was performed according to the manufacturer’s instructions (10X Genomics) and as described previously (88). Briefly, each cell was partitioned, along with a single barcoded Gel Bead into an individual Gel Bead in Emulsion (GEM). Once the Gel Bead dissolved and the cell was lysed, a reverse transcription reaction allowed the cell to be individually and uniquely barcoded. The barcoded cDNA was then amplified with primers specific to Ig-constant regions, resulting in targeted enrichment of the V(D)J. The V(D)J enrichment kit included all reagents and primers for PCR amplification of the full-length variable and constant regions. This process resulted in a library of full-length Ig genes ready for next-generation sequencing.

### Next-generation sequencing.

The amplified transcripts were sequenced using a NovaSeq S1 System (Illumina) by the Oklahoma Medical Research Foundation Clinical Genomics Center. Libraries were sequenced to a depth of 200 million paired reads. The raw output files were processed using Cell Ranger 3.0.2 software (10X Genomics) to generate FASTA files for further analysis. For the CTD^+^ Bmem cells, this resulted in 2262, 4394, and 1826 annotated heavy and light chain sequences for subjects 1008, 1009, and 1013, respectively. For the CTD^–^ Bmem cells, this resulted in 16684, 16009, and 15868 annotated sequences for subjects 1008, 1009, and 1013, respectively. These sequences were further curated for full-length, productive sequences that had a single heavy and light chain pair. This curation resulted in 506, 948, and 527 CTD^+^ Bmem sequences and 4443, 4612, and 3845 CTD^–^ Bmem sequences used in this study for subjects 1008, 1009, and 1013, respectively. Difference in frequency of sequences between 1009 and other subjects was less than 2-fold for both CTD^+^ and CTD^–^ Bmem cells, allowing appropriate comparisons.

### RNA-seq data analysis.

The FASTA files were uploaded to the IMGT HighVQuest website (http://imgt.org/HighV-QUEST/home.action). Data from IMGT HighVQuest were parsed in RStudio (http://www.rstudio.com/) using MakeDb.py from Change-O (89). These data were then filtered to keep barcodes associated with sequences that were functional, full-length, and had only 1 heavy chain and light chain pair. Clones were defined and germlines assigned with DefineClones.py and CreateGermlines.py, respectively, from Change-O using the hamming distance algorithm. To assign clonally related sequences, a clustering threshold of 85% (CDR3 sequence similarity) was determined with distToNearest function and findThreshold function. Mutations in the heavy chain V regions were calculated using observedMutations function from the SHazaM R package (89).

### Human mAb synthesis.

Genes for mAb generation were selected from the IgG1 sequences from CTD^+^ Bmem cells (Supplemental Table 2). To limit selection bias, sequences that spanned various heavy chain CDR3 lengths, numbers of V region mutations, and V region usage were included. The mAbs were generated using a previously published method (90), with the exception of using sequences generated using 10X Genomics technology rather than single-cell reverse transcription PCR (RT-PCR). To express mAbs from CTD^+^IgM^+^ Bmem cells from each subject, we selected sequences belonging to clonal families with several members (subject 1008, 4 members; 1009, 8, 9, and 11 members; 1013, 6 members). The heavy chain V region from these sequences were expressed with an IgG1 heavy chain constant region. After deep sequencing, heavy and light chain genes were synthesized by IDT (Integrated DNA Technologies). These genes were then cloned into separate heavy and light chain (κ or λ as appropriate) vectors and transformed using DH5α competent cells (Invitrogen). Four colonies were picked from the transformation for mini-prep followed by sequencing. The sequences from each of the colonies were matched to the consensus, and the best match was moved forward to maxi-scale preparation. Human kidney epithelial cells (HEK293 cell line; ATCC) were transiently cotransfected with heavy and light chain vectors using polyethylenimine, and the cells were allowed to produce Abs over 5 days. Pierce Protein A agarose beads (Thermo Fisher Scientific) were used to purify the mAb from the cell culture supernatant. The mAbs were then analyzed for concentration, specificity, and biological activity.

### ELISA.

Maxisorp (Thermo Fisher Scientific) plates were coated with 100 μL/well of CTD (10 μg/mL) diluted in carbonate coating buffer and incubated at 4°C overnight. After washing with PBS/0.05%Tween-20, plates were blocked with 200 μL/well PBS/0.05%Tween-20/1%BSA (blocking buffer) for 2 hours at room temperature. After washing the plates, 100 μL/well of mAbs diluted 10 μg/mL in blocking buffer were added and allowed to incubate for 2 hours at room temperature. After 4 washes, HRP-anti human IgG (Jackson ImmunoResearch, 1:2500 in blocking buffer) was added to plates and incubated for 1 hour at room temperature. ABTS was added to develop the plates, and stop buffer (10% SDS w/v ddH_2_O) was added to stop the reaction after 5 minutes. OD was measured at 405 nm. For some of the mAbs, individual ELISA curves generated from a series of sixteen 2-fold dilutions of the Ab starting at 10 μg/mL were applied to a curve-fitting analysis to calculate Ab affinities (*K_D_*) as described (91).

### In vitro neutralization assay.

CHO cells were resuspended at 1 × 10^5^ cells/mL in F-12K media (Corning) with L-glutamine and 10% heat-inactivated FCS, and 100 μL of the cell suspension was seeded into each well of the 96-well plates and cultured overnight (5% CO_2_, 37°C). Human mAbs and plasma samples were diluted to 10 μg/mL and 1/100, respectively, in medium containing TcdB1 or TcdB2 at a final concentration of 0.23 nM and 0.25 nM, respectively, and incubated for 30 minutes at 37°C. A positive control serum was obtained from rabbits immunized and boosted with 0.1 mg of the CTD fraction of *C*. *difficile* VPI-10463 ribotype as described previously (57). IgG depletion from plasma samples was achieved using albumin and IgG Depletion SpinTrap columns (GE Life Sciences) prepacked with agarose beads covalently bound to anti-HSA and Protein G. The toxin concentration was calibrated to cause 80% CHO cell death. Medium from 24-hour-old cultures was removed and replaced with the Ab/plasma-toxin-medium mixture. Plates were cultured for 24 hours before addition of 100 μL/well of media containing 10% Cell Counting kit-8 (CCK-8) reagent (MilliporeSigma) and an additional 2 hours of incubation. Absorbance at a wavelength of 450 nm (A450) was then measured, and percent viability was calculated as follows: (A450 of treated [sample-toxin] cells/A450 of untreated [no toxin] cells) × 100.

### Dataset availability.

Sequences are submitted to the NCBI Sequence Read Archive (SRA), and the project is registered with the BioProject database under SRA accession PRJNA601978 (http://www.ncbi.nlm.nih.gov/bioproject/601978). The accession nos. for the data are: SAMN13878386 (subject 1007), SAMN13878387 (subject 1008), SAMN13878388 (subject 1009) and SAMN13878389 (subject 1013).

### Statistics.

GraphPad Prism software was used to generate binding curves and calculate equilibrium *K_D_* to report mAb affinity. For multiple comparisons Kruskal-Wallis with Dunn’s post hoc test correction was used as indicated. A 2-tailed unpaired *t* test with Welch’s correction was used to compare 2 groups. *P* < 0.05 was considered significant.

### Study approval.

Written informed consent was given by study participants. The human subject studies were approved by the OUHSC IRB (protocol no. 8158) and were performed in accordance with the ethical standards as laid down in the 1964 Declaration of Helsinki and its later amendments.

## Author contributions

HBS designed and performed the experiments, analyzed data, and wrote the manuscript. MLL devised the project, analyzed data, and wrote the manuscript. EJS performed bioinformatics analysis and curated the data. KS generated and quality control–checked the mAbs, analyzed data, and edited the manuscript. JLL provided critical reagents and assisted with neutralization experiments. JDB provided critical reagents, reviewed data, and edited the manuscript. JAJ assisted with subject recruitment, reviewed data, and edited the manuscript.

## Supplementary Material

Supplemental data

## Version 1 07/14/2020

In-Press Preview

## Version 2 08/20/2020

Electronic publication
