# Supplementary material for: Human C. difficile toxin–specific memory B cell repertoires encode poorly neutralizing antibodies
Source: JCI Insight. 2020 Aug 20;5(16):e138137. doi: 10.1172/jci.insight.138137 (PMC7455132; doi:10.1172/jci.insight.138137)
Supplement: Supplemental data [file jciinsight-5-138137-s011.pdf]

SUPPLEMENTAL METHODS

Polyclonal stimulation of B_{mem} cells and ELISPOT. PBMCs were re-suspended in RPMI 1640 media containing 10% FCS, 2mM L-glutamine and 1% antibiotic-antimycotic solution (complete culture media) with polyclonal stimuli to differentiate B_{mem} cells into Ab-secreting plasma cells. Briefly, CpG 2006 (Invivogen, San Diego, CA) at 6 µg/ml, Pokeweed Mitogen (a kind gift from Emory University Vaccine Center) at 1/100,000 dilution of stock, and Staphylococcus aureus protein A (Sigma, St. Louis, MO) at 1/10,000 dilution of stock were added to PBMCs seeded at 0.5×10^6 cells in 0.5 mL per well in 24-well plates and cultured for 6 days. Cells were collected from the 24-well plates on day 6, washed and re-suspended in complete culture media. One day before the end of the culture period, hydrophobic high protein binding Immobilon-P multiscreen plates (Millipore, Bedford, MA) were coated with PBS containing final concentrations of 10 µg/mL CTD and 10 µg/mL goat anti-human IgG (Jackson Immunoresearch, West Grove, PA). Plates were then washed and blocked with 10% FCS in RPMI 1640 before addition of cultured cells. Cells were titrated such that 2×10^6 or 0.5×10^6 cells were added per well and serially diluted as indicated. After 5 hr incubation (37°C, 5% CO2), cells were removed by lysis in PBS/0.05% Tween 20 and plates were washed. Horseradish peroxidase (HRP)-conjugated anti-human IgG and IgM (Southern Biotech, Birmingham, AL) diluted to 1/1000 and 1/2500 of the stock respectively were added to detect Ab-secreting cells. Following an overnight incubation, plates were developed using AEC substrate (Sigma, St. Louis, MO). After spots developed, plates were washed 20 times with ddH_2O before allowing plates to dry. Spots were scanned and enumerated using the Immunospot software (Cellular Technology Ltd., Cleveland, OH).

SUPPLEMENTAL FIGURE LEGENDS

Supplemental Figure 1. Detection of CTD^+ and CTD^- B_{mem} cells. (A) Depicts the full gating strategy utilized to isolate CTD^+ and CTD^- B_{mem} cells from enriched peripheral blood B cells. Pseudocolor plots 1 through 7 depict the full gating strategy allowing identification of Single cell, $CD3^-$, $CD19^+$, $CD20^+$, $CD27^+$, $CD38^-$ (B_{mem} cells), CTD^+ and CTD^- cells. Data from subject 1013 is shown.(B) Detection of peripheral blood CTD^+ B_{mem} cells in subject 1009 was done as in (A).

Supplemental Figure 2. Specificity and function of CTD^+ and CTD^- B_{mem} cells. (**A**) PBMCs from subject 1018 were labeled as described in the methods except that a goat anti-human IgA, IgM and IgG (BCR-blocking) Ab was added before the fluorophore-conjugated mAbs. Pseudocolor plots 1 through 3 depict the ability to detect CTD^- and CTD^+ B_{mem} cells in the absence (top row) and presence (bottom row) of BCR-blocking Ab. (B) PBMCs isolated from six subjects with a history of CDI (including 1008, 1009 and 1013) and four healthy controls were cultured with polyclonal stimuli to drive differentiation of B_{mem} cells to antibody-secreting plasmablast cells (ASC). Cells were then added to multiscreen plates to detect total IgG (left) and CTD-specific (right) IgG-secreting cells as described in the supplemental methods. The number of ASC detected per million PBMCs is indicated on the graphs. The line indicates the mean. Volunteers 1008, 1009, and 1013 had 11.5, 1, and 0 spots /10^6 ASC respectively.

Supplemental Figure 3. ELISPOT analysis detects predominantly IgM^+ CTD^+ Bmem cells. PBMCs from subject 1018 were stimulated or not *in vitro* as described in the supplemental methods. The images with CTD-specific spots were from wells loaded with one million cells. Wells for total IgM and IgG were loaded with 0.25 million cells. Error bars in graphs represent S.E.M for duplicate samples.

Supplemental Figure 4. VH3-JH gene pairing in Bmem cells from subjects 1009 and 1013. Heat maps depict the VH3-JH gene recombination pairs for the CTD^{+} Bmem cell and the CTD^{-} Bmem cells from subjects (A) 1009 and (B) 1013. The color scale indicates the frequency of occurrence of each VH3-JH pair. Pale yellow represents a frequency of zero and blue represents frequencies above zero.

Supplemental Figure 5. Bmem Ab repertoire from an individual with no known history of *C. difficile* infection. (A) Plots show gating strategy for Bmem cell (CD3^{-}/CD20^{+}/CD19^{+}/CD27^{+}/CD38^{-}) sorting by flow cytometry. B cells were enriched from a whole blood sample from a healthy control subject and IgM^{+} and IgM^{-} Bmem were isolated as depicted by pseudocolor plots 1 to 3. (B) Depicts IgG subclass distribution within the IgM^{-} Bmem cells. (C) Shows V gene usage in heavy chain sequences from IgM^{+} (left) and IgM^{-} Bmem (right). (D) Depicts the percent replacement and silent nucleotide mutations in the heavy chain V regions of IgA, IgG and IgM sequences as compared to germline. A Kruskal-Wallis test with Dunn's post-test correction was used to determine statistical significance in differences between mutation frequencies for each Ab isotype (**** , $p < 0.0001$).

Supplemental Figure 6. Bmem Ab repertoire from an individual with no known history of *C. difficile* infection. (A) Depicts the number of IgG1 heavy chain sequences with the indicated range of nucleotide changes, as compared to germline sequences. Each mutation recorded resulted in an amino acid change (replacement mutation). (B) Depicts the amino acid length distribution of the CDR3 region in IgG1 heavy chain sequences of the IgM^{-} Bmem cells. (C) Depicts clonal diversity in the IgM^{+}, IgG^{+} and IgA^{+} Bmem cells. The number in the center of each chart denotes the number of sequences analyzed. The numbers in the legends to the right of

each chart indicate the size of a given clone. The shaded areas represents the frequency with which clones of each size appeared within the total sample.

Supplemental Figure 7. Non-neutralizing mAbs generated from expanded IgM clones. V(D)J sequences from expanded IgM clones from subjects 1008, 1009, and 1013 were used to generate mAbs with IgG1 constant regions. (A) The CTD-binding capacity of these mAbs was tested by ELISA and (B) their capacity to neutralize TcdB1 *in vitro*. The neutralization experiments were performed twice with the same results. The error bars represent S.E.M. for triplicate samples.

Supplemental Table 1. Demographics and disease status of Individuals included in this study. [A]Control denotes a healthy individual with no previous history of *C. difficile* infection. [B]Subject refers to a currently healthy individual with a previous history of *C. difficile* infection. [C]Time refers to the interval between infection and sample procurement.

Supplemental Table 2. Heavy chain VDJ genes and CDR3 amino acid sequences of the mAbs generated from subjects 1008, 1009 and 1013. Unfilled rows depict IgG1 sequences. Shaded rows depict IgM sequences.

(A) Full gating strategy pre-sort for enriched B cells from volunteer 1013

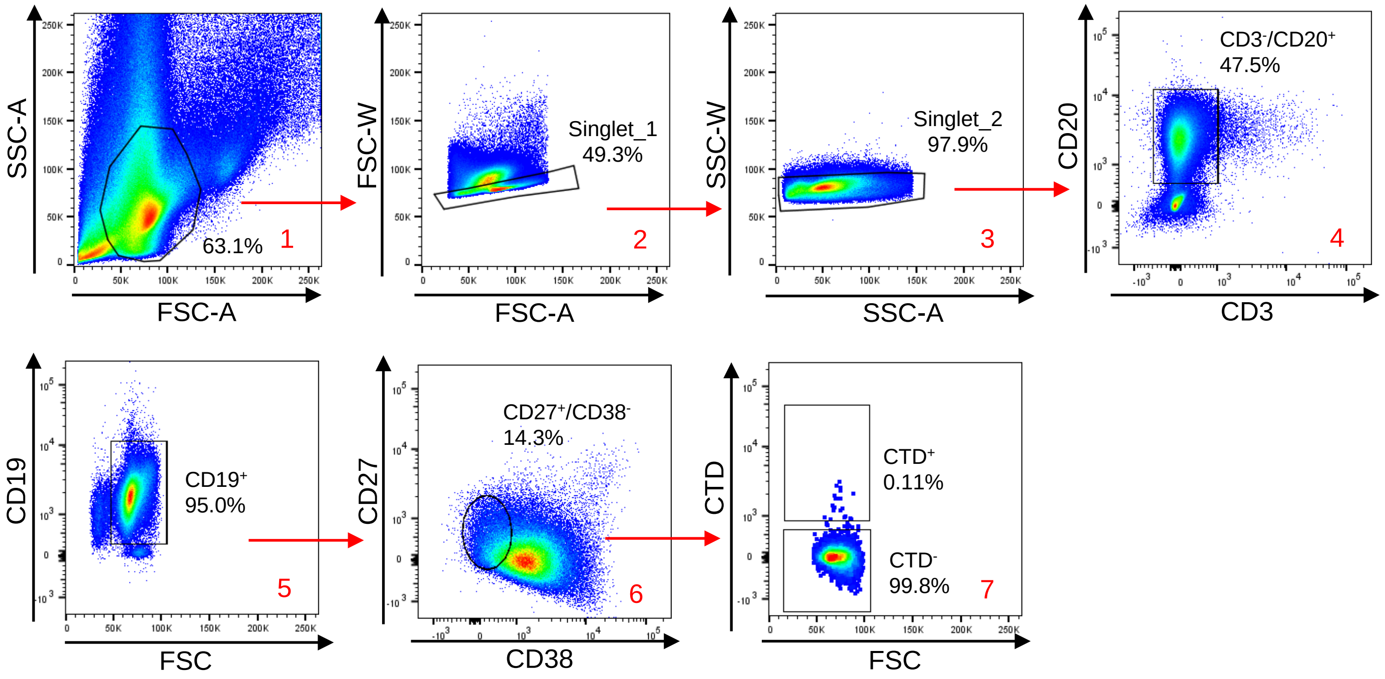

(B) CTD+ and CTD- Bmem sorting strategy for volunteer 1009

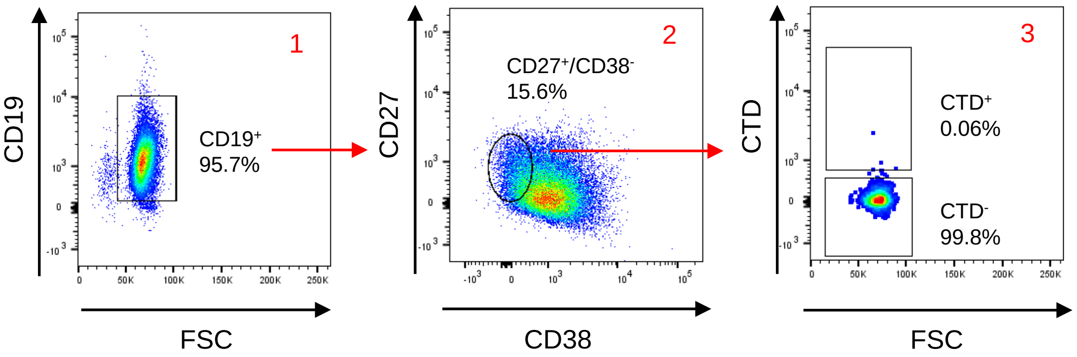

Supplemental Figure 1. Detection of CTD$^+$ and CTD$^-$ Bmem cells. (A) Depicts the full gating strategy utilized to isolate CTD$^+$ and CTD$^-$ Bmem cells from enriched peripheral blood B cells. Pseudocolor plots 1 through 7 depict the full gating strategy allowing identification of Single cell, CD3$^-$, CD19$^+$, CD20$^+$, CD27$^+$, CD38$^-$ (Bmem cells), CTD$^+$ and CTD$^-$ cells. Data from subject 1013 is shown.(B) Detection of peripheral blood CTD$^+$ Bmem cells in subject 1009 was done as in (A).

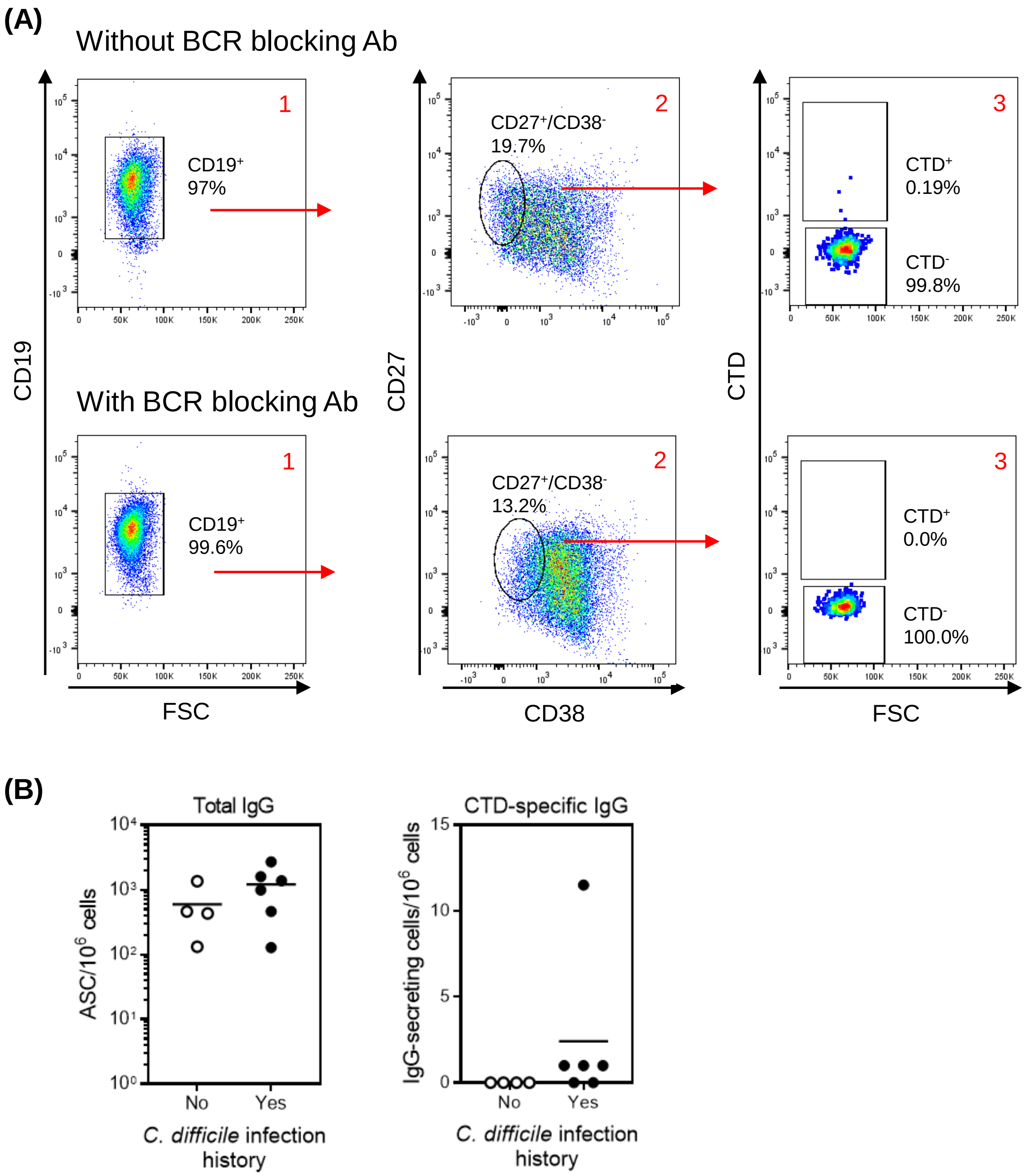

Supplemental Figure 2. Specificity and function of CTD+ and CTD- Bmem cells. (**A**) PBMCs from subject 1018 were labeled as described in the methods except that a goat anti-human IgA, IgM and IgG (BCR-blocking) Ab was added before the fluorophore-conjugated mAbs. Pseudocolor plots 1 through 3 depict the ability to detect CTD- and CTD+ Bmem cells in the absence (top row) and presence (bottom row) of BCR-blocking Ab. (B) PBMCs isolated from six subjects with a history of CDI (including 1008, 1009 and 1013) and four healthy controls were cultured with polyclonal stimuli to drive differentiation of Bmem cells to antibody-secreting plasmablast cells (ASC). Cells were then added to multiscreen plates to detect total IgG (left) and CTD-specific (right) IgG-secreting cells as described in the supplemental methods. The number of ASC detected per million PBMCs is indicated on the graphs. The line indicates the mean. Volunteers 1008, 1009, and 1013 had 11.5, 1, and 0 spots /10^6 ASC respectively.

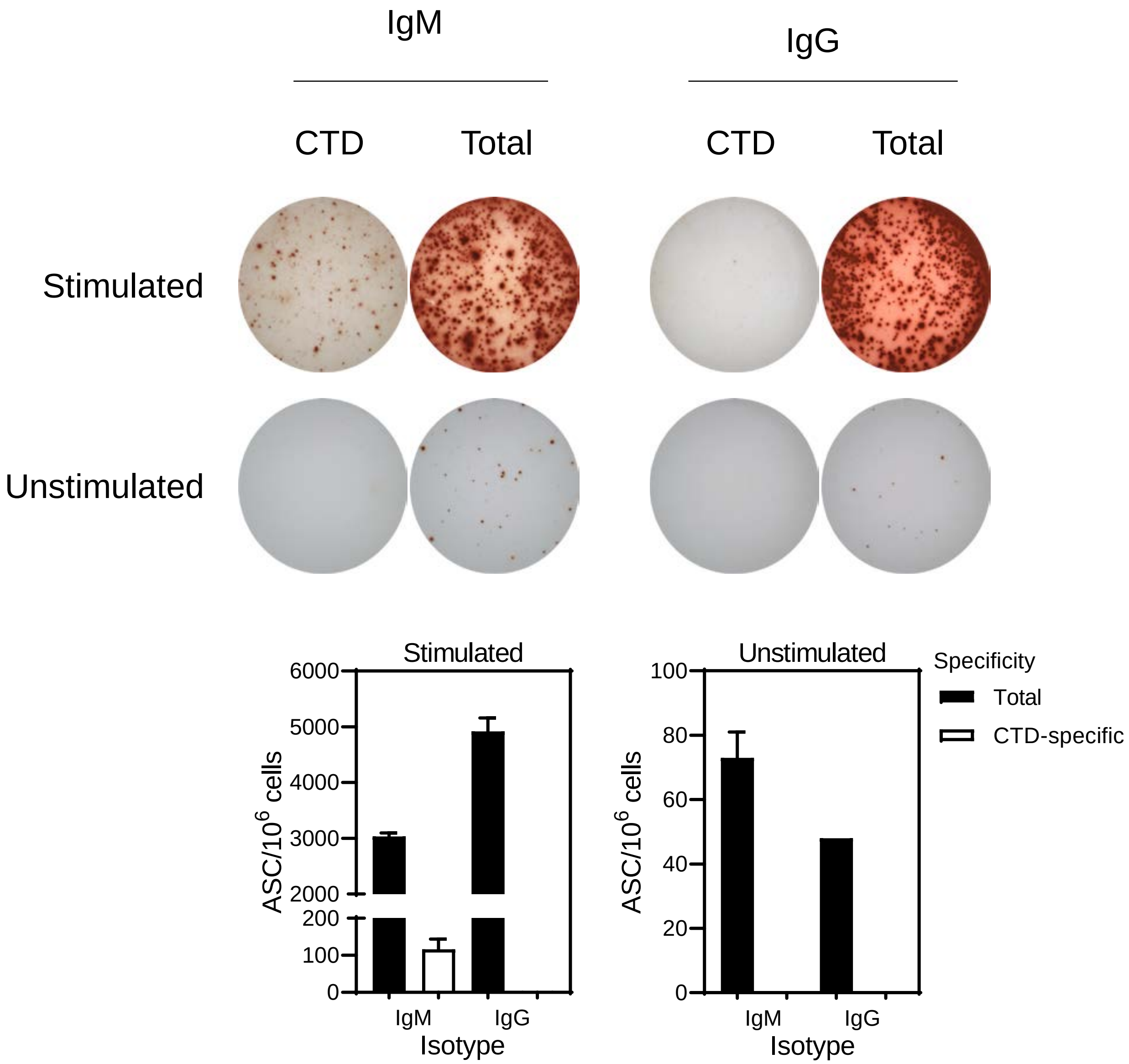

Supplemental Figure 3. ELISPOT analysis detects predominantly IgM⁺ CTD⁺ Bmem cells. PBMCs from subject 1018 were stimulated or not *in vitro* as described in the supplemental methods. The images with CTD-specific spots were from wells loaded with one million cells. Wells for total IgM and IgG were loaded with 0.25 million cells. Error bars in graphs represent S.E.M for duplicate samples.

(A) 1009

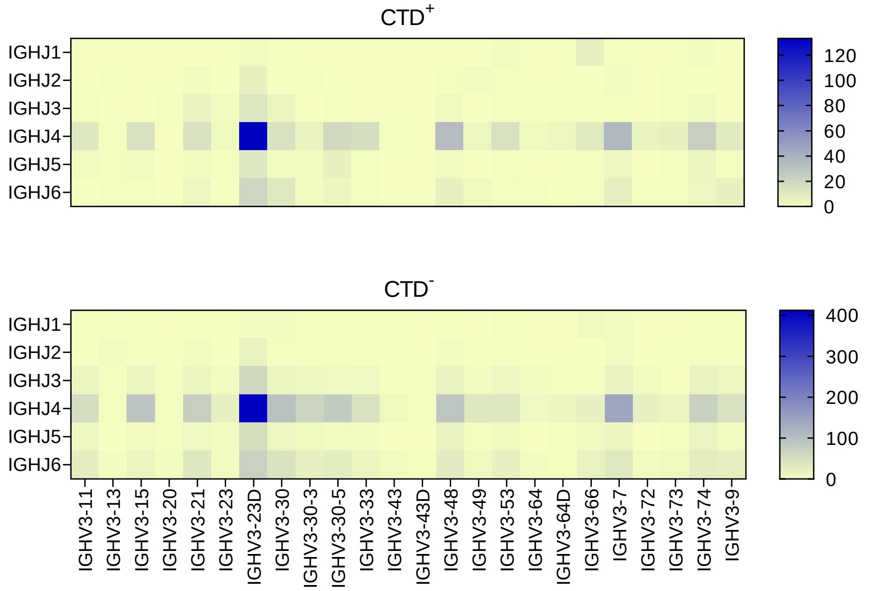

(B) 1013

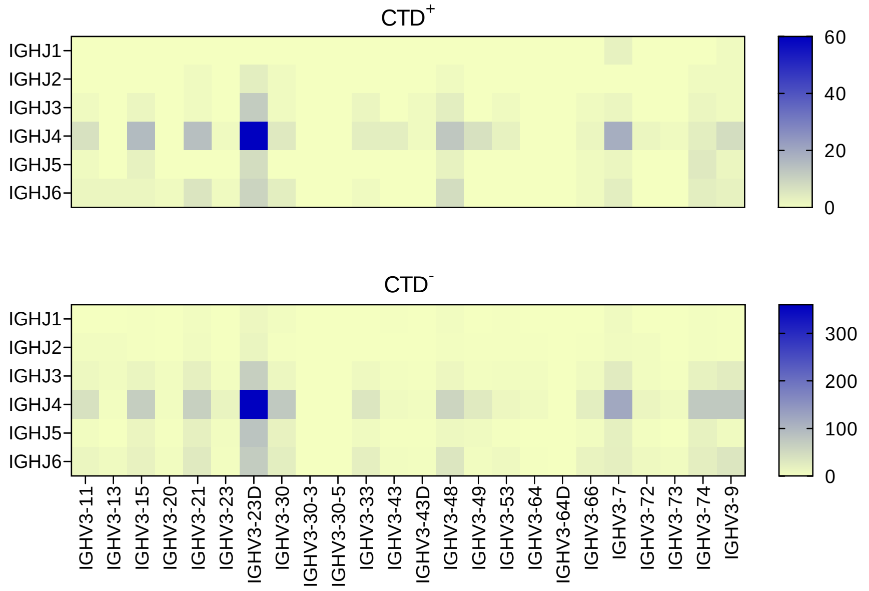

Supplemental Figure 4. VH3-JH gene pairing in Bmem cells from subjects 1009 and 1013. Heat maps depict the VH3-JH gene recombination pairs for the CTD^{+} Bmem cell and the CTD^{-} Bmem cells from subjects (A) 1009 and (B) 1013. The color scale indicates the frequency of occurrence of each VH3-JH pair. Pale yellow represents a frequency of zero and blue represents frequencies above zero.

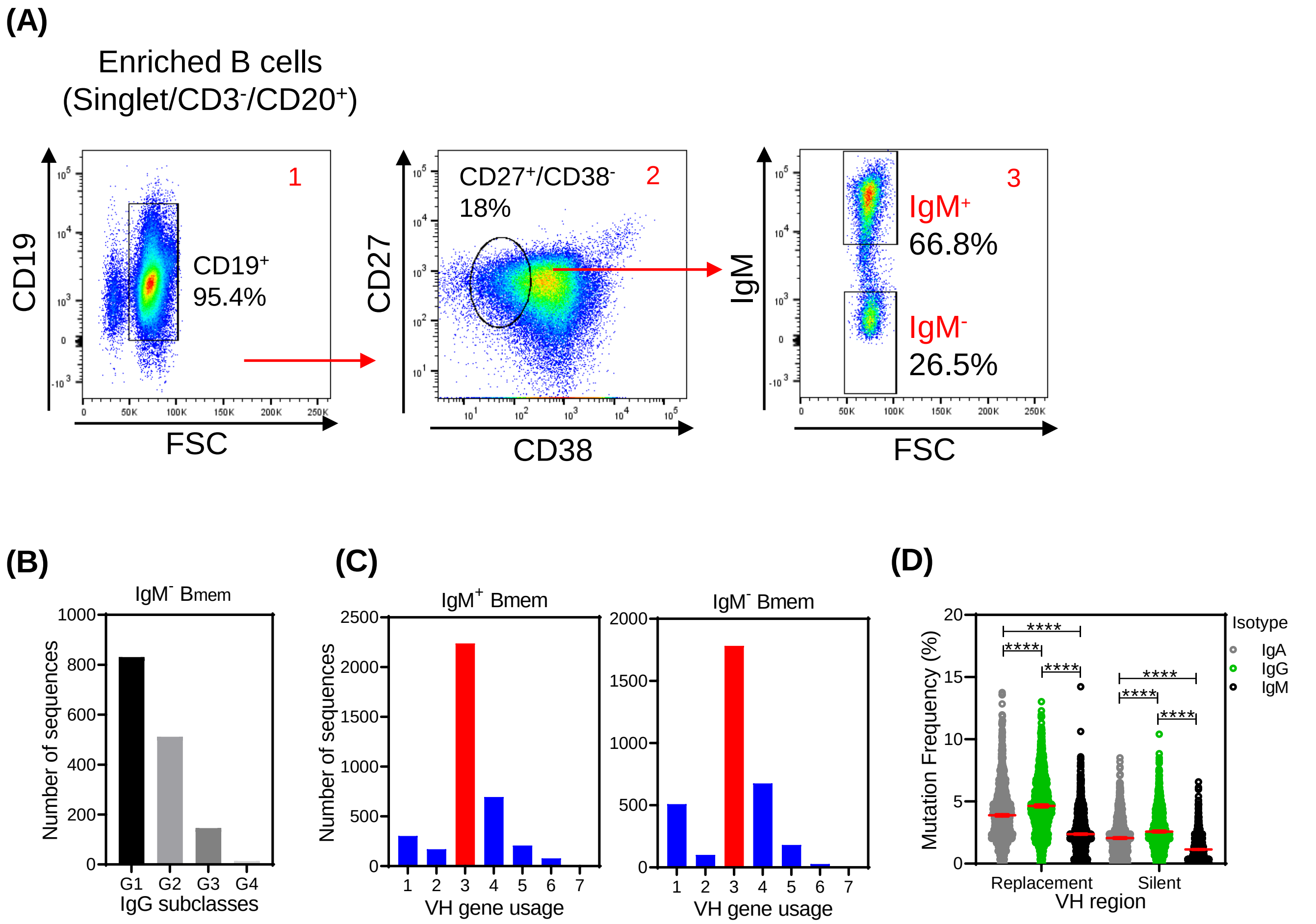

Supplemental Figure 5. Bmem Ab repertoire from an individual with no known history of *C. difficile* infection. (A) Plots show gating strategy for Bmem cell ($CD3^-/CD20^+/CD19^+/CD27^+/CD38^-$) sorting by flow cytometry. B cells were enriched from a whole blood sample from a healthy control subject and IgM^+ and IgM^- Bmem were isolated as depicted by pseudocolor plots 1 to 3. (B) Depicts IgG subclass distribution within the IgM^- Bmem cells. (C) Shows V gene usage in heavy chain sequences from IgM^+ (left) and IgM^- Bmem (right). (D) Depicts the percent replacement and silent nucleotide mutations in the heavy chain V regions of IgA, IgG and IgM sequences as compared to germline. A Kruskal-Wallis test with Dunn's post-test correction was used to determine statistical significance in differences between mutation frequencies for each Ab isotype (**** , p <0.0001).

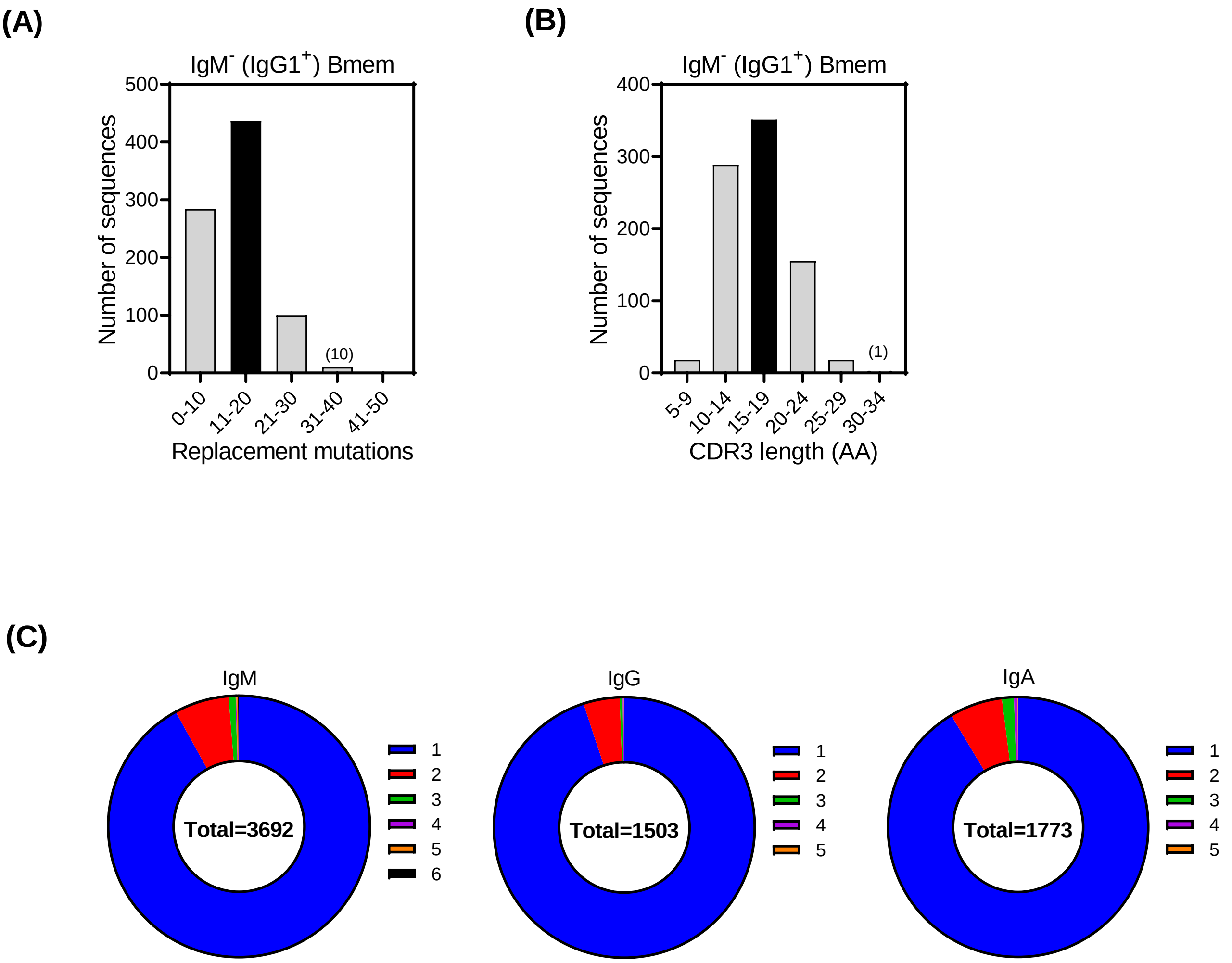

Supplemental Figure 6. Bmem Ab repertoire from an individual with no known history of *C. difficile* infection. (A) Depicts the number of IgG1 heavy chain sequences with the indicated range of nucleotide changes, as compared to germline sequences. Each mutation recorded resulted in an amino acid change (replacement mutation). (B) Depicts the amino acid length distribution of the CDR3 region in IgG1 heavy chain sequences of the IgM$^-$ Bmem cells. (C) Depicts clonal diversity in the IgM$^+$, IgG$^+$ and IgA$^+$ Bmem cells. The number in the center of each chart denotes the number of sequences analyzed. The numbers in the legends to the right of each chart indicate the size of a given clone. The shaded areas represents the frequency with which clones of each size appeared within the total sample.

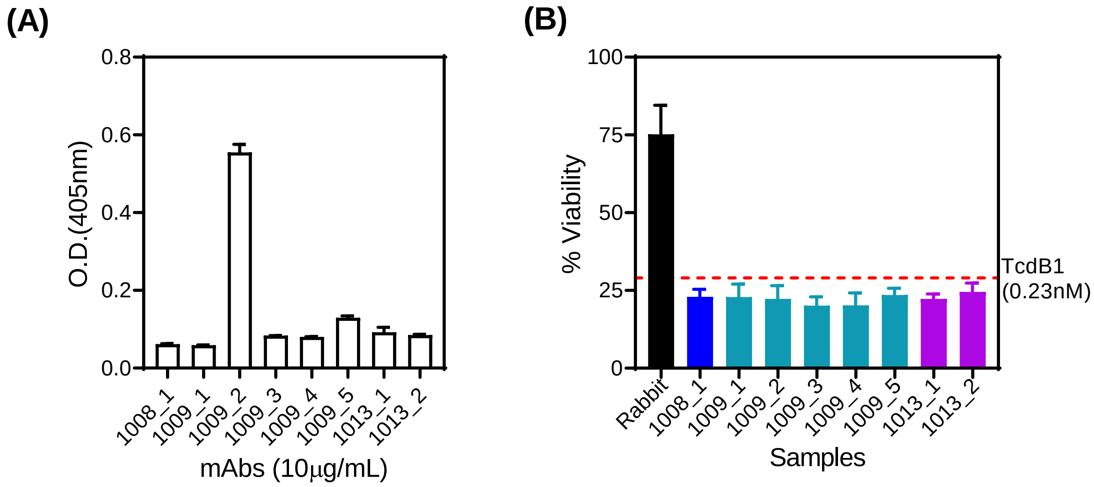

Supplemental Figure 7. Non-neutralizing mAbs generated from expanded IgM clones. V(D)J sequences from expanded IgM clones from subjects 1008, 1009, and 1013 were used to generate mAbs with IgG1 constant regions. (A) The CTD-binding capacity of these mAbs was tested by ELISA and (B) their capacity to neutralize TcdB1 *in vitro*. The neutralization experiments were performed twice with the same results. The error bars represent S.E.M. for triplicate samples.

	Age (yrs)	Gender	Race	Status	Time[C] (months)	Medication
1007	56	Female	Caucasian	Control[A]	N/A	N/A
1008	41	Female	African American	Subject[B]	18	Metronidazole
1009	26	Female	Caucasian	Subject	27	Information unavailable
1013	30	Female	Caucasian	Subject	32	Metronidazole + Vancomycin
1018	43	Male	Caucasian	Subject	8	Information unavailable

Supplemental Table 1. Demographics and disease status of Individuals included in this study. [A]Control denotes a healthy individual with no previous history of *C. difficile* infection. [B]Subject refers to a currently healthy individual with a previous history of *C. difficile* infection. [C]Time refers to the interval between infection and sample procurement.

mAbs	V gene	D gene	J gene	CDR3 (AA)
1008_05	IGHV4-39	IGHD4-23	IGHJ4	ARRPRDHRSPKHYFDY
1008_11	IGHV3-66	IGHD3-16	IGHJ3	ARSRVQGQIVNDAFDL
1008_13	IGHV3-30-3	IGHD5-24	IGHJ6	AREVNDWVQLQSPYFYYGMDV
1008_19	IGHV4-34	IGHD2-2	IGHJ1	ARGFRTTGWYGPQSFPH
1008_20	IGHV1-69	IGHD5-24	IGHJ6	ARAPSGPGRIETVAEDYHYQGMDV
1008_21	IGHV3-49	IGHD3-3	IGHJ4	SRSGTDNNFWSGYSDS
1008_22	IGHV1-46	IGHD4-17	IGHJ2	ARDQSSSGTASWFSDL
1008_24	IGHV4-39	IGHD2-8	IGHJ4	CAGSYCRDGVCYGHVEDLFDY
1008_25	IGHV2-5	IGHD3-3	IGHJ5	AHTGYDFWSGYPEHENWFEP
1008_26	IGHV3-23	IGHD3-10	IGHJ4	AKDRGITMIRGLITPFDY
1008_29	IGHV3-66	IGHD3-22	IGHJ4	ARVSSGERGKPMIYDSSGYLDY
1008_30	IGHV2-5	IGHD5-24	IGHJ4	VHRDGNYLNPRSYSFDY
1008_31	IGHV3-23	IGHD2-2	IGHJ4	NPTQQHQPSAFHY
1008_35	IGHV3-9	IGHD4-23	IGHJ4	VKGLLLYGAKSKSNHFDS
1008_38	IGHV5-51	IGHD6-25	IGHJ4	ARYATVMKRVDY
1008_43	IGHV2-70	IGHD6-6	IGHJ6	ARIPFSRDADDDPRGGRRIHHGVDV
1008_IgM	IGHV3-43	IGHD6-13	IGHJ3	AKMEEQQLNAFDI
1009_01	IGHV3-9	IGHD3-22	IGHJ4	AKDTNWYDTRGYDGALES
1009_02	IGHV3-30	IGHD2-15	IGHJ6	AREDDCSGGGCYGMDV
1009_03	IGHV3-7	IGHD5-18	IGHJ6	ARGTPWGEYTYQSPYYYYGMDV
1009_04	IGHV1-8	IGHD4-11	IGHJ3	ATTYNNDDGFDI
1009_05	IGHV1-46	IGHD3-3	IGHJ4	ARDQGTYSNFWSGYYPDY
1009_06	IGHV4-39	IGHD3-10	IGHJ5	ARLRAIMLRGVVAPAWFDT
1009_07	IGHV2-26	IGHD6-19	IGHJ4	ARMRGGWNYFDY
1009_08	IGHV3-74	IGHD4-23	IGHJ6	ARGTEGPEGYDYYGMDV
1009_09	IGHV4-34	IGHD6-6	IGHJ2	ARDPPISTSSVGRSGSPRKTTTTSYWYFDL
1009_10	IGHV1-2	IGHD3-3	IGHJ5	ARDRDFWSGYFLGSSQKNCLDP
1009_11	IGHV1-2	IGHD3-22	IGHJ6	ARSSRENPDRSGTLSWGPVGRQYHYYFGLDV
1009_12	IGHV1-69	IGHD3-16	IGHJ4	ARGTDDYVWGAYRTDLDY
1009_14	IGHV3-23	IGHD3-22	IGHJ4	AKSLASSSLNHYDSRGYLGGFDY
1009_15	IGHV3-15	IGHD6-6	IGHJ4	TAEVRVHSDSSSLFEY

mAbs	V gene	D gene	J gene	CDR3 (AA)
1009_16	IGHV1-18	IGHD6-19	IGHJ4	ARDVRKHSSGWSPFAY
1009_17	IGHV3-23	IGHD5-18	IGHJ4	AKAVLGYSFGAKYYFDY
1009_18	IGHV5-51	IGHD3-22	IGHJ3	ARLAGDGTGYYYPLGGDAFDI
1009_19	IGHV3-74	IGHD3-22	IGHJ1	VRGGRSYDSGGYYSAEYFQH
1009_20	IGHV3-49	IGHD6-19	IGHJ6	SRALSRGWYSPDDYYSGLDV
1009_IgM_01	IGHV3-66	IGHD2-21	IGHJ1	VSGYCGGDCLYFQH
1009_IgM_02	IGHV6-1	IGHD3-22	IGHJ4	AGAVSSGYYHFDH
1009_IgM_03	IGHV4-59	IGHD5-18	IGHJ4	ARAIRGYSYVFGY
1009_IgM_04	IGHV3-23	IGHD2-8	IGHJ4	AKDQGYCINNVCYFSSSRSDY
1009_IgM_05	IGHV3-23	IGHD2-8	IGHJ4	AKDQGYCTNGICYFSSSRSDY
1013_01	IGHV4-34	IGHD3-3	IGHJ4	TRVAYTFWSGYSYYFDN
1013_02	IGHV1-18	IGHD1-26	IGHJ4	ARAQVPVGTLSPDY
1013_03	IGHV1-69	IGHD2-8	IGHJ6	ARDGLDIGVMEYYNGMDV
1013_04	IGHV3-11	IGHD2-8	IGHJ4	ARDQLGILPDY
1013_05	IGHV3-13	IGHD5-12	IGHJ6	VRGIVSTLSSYYMDV
1013_06	IGHV3-15	IGHD3-22	IGHJ3	ATTTKNLGDAFDL
1013_07	IGHV3-23	IGHD1-26	IGHJ6	AKWGSSGNYGLNYYYFYGLDV
1013_08	IGHV3-23	IGHD2-2	IGHJ6	VKDRAYQLLGVYYHHNMDV
1013_09	IGHV3-30	IGHD5-12	IGHJ3	AKGRYTWLRLGNALDI
1013_10	IGHV3-7	IGHD5-24	IGHJ1	ATSIGNGYNLGISFQS
1013_11	IGHV3-9	IGHD2-21	IGHJ4	AKDLIEGRRSLGFDS
1013_12	IGHV4-39	IGHD6-13	IGHJ4	ARLGIHAVVDF
1013_13	IGHV4-59	IGHD2-15	IGHJ2	ARGGYCSGGGCYSRPDWYFDL
1013_14	IGHV5-51	IGHD4-17	IGHJ6	ARLTSALDRDLEGHPYYYYMDV
1013_IgM_01	IGHV1-2	IGHD3-22	IGHJ4	CARDSETSGYQFDYW
1013_IgM_02	IGHV3-48	IGHD6-19	IGHJ6	CARARMAVAGYYYYMDVW

Supplemental Table 2. Heavy chain VDJ genes and CDR3 amino acid sequences of the mAbs generated from subjects 1008, 1009 and 1013. Unfilled rows depict IgG1 sequences. Shaded rows depict IgM sequences.
